# The Associations Between Habitual Dietary Fat Intake and Inflammatory Markers Among Marathon Runners: An Exploratory Study

**DOI:** 10.3390/nu18142273

**Published:** 2026-07-11

**Authors:** Qi Jin, David Aguilar, Damon Joyner, Stacie Wing-Gaia, Saori Hanaki, Bryan Dowdell, Jamie Stein

**Affiliations:** Department of Exercise and Nutrition Sciences, Dumke College of Health Professions, Weber State University, Suit 302, Swenson Building, 1435 Village Dr., Ogden, UT 84408, USA

**Keywords:** nutrition, sports nutrition, marathon performance, marathon recovery, inflammation, males and females, dietary fat quality, fatty acid

## Abstract

Background: Marathon running triggers a robust systemic inflammatory response. Whether habitual dietary fat composition modulates pre-race cytokine concentrations or the magnitude and recovery of the exercise-induced inflammatory response remains unknown. Methods: Thirty-one recreational marathon runners (58.1% female; mean age 38.4 ± 10.2 years) completed the Diet History Questionnaire III to assess habitual dietary fat intake—including chain-length-specific SFA, MFA, PFA, trans fatty acids, and CLA—over the preceding three months. Circulating IFN-γ, IL-1β, IL-4, IL-6, IL-10, and TNF-α were measured pre-race, immediately post-race, and 48 h post-race. Repeated measures ANOVA examined cytokine changes; multivariable regression models estimated fat–cytokine associations adjusted for 11 pre-specified covariates, with Benjamini–Hochberg FDR correction applied across all models. Results: IL-6 rose markedly post-race (*p* < 0.001, generalized η^2^ = 0.615) before returning to pre-race levels by 48 h. IL-10 followed a similar trajectory (*p* < 0.001, generalized η^2^ = 0.346). TNF-α showed a borderline non-significant trend (*p* = 0.056); IFN-γ, IL-1β, and IL-4 did not change significantly. MFA 20:1 was positively associated with IFN-γ concentrations at 48 h post-race (β = 13.66; 95% CI: 8.94, 18.38, *p* < 0.01, q = 0.04) and TNF-α (β = 9.14; 95% CI: 6.43, 11.86, *p* < 0.01, q = 0.02). Conclusions: Habitual intake of the long-chain monounsaturated fatty acid 20:1 was positively associated with IFN-γ and TNF-α at 48 h post-race. This pattern, emerging specifically at the 48 h post-race phase, suggests that the post-race period, once acute exercise-induced signaling has subsided, may be a window in which habitual dietary fat quality is most detectable in relation to circulating cytokines. Habitual fat quality may warrant consideration in future endurance recovery nutrition research. This exploratory finding calls for replication in larger cohorts.

## 1. Introduction

The number of recreational non-elite marathon runners have grown substantially [1,2]. This population is characterized by wide heterogeneity in training histories and dietary habits [3,4]. The evidence for managing the physiological consequences of marathon participation regarding acute systemic inflammatory response remains underdeveloped [4,5]. Marathon running elicits an acute, transient, systemic inflammatory response driven by mechanical muscle microtrauma, and metabolic and oxidative stress [6]. Circulating interleukin-6 (IL-6) rises markedly following marathon running [7,8]. This IL-6 surge is accompanied by a parallel elevation in the counter-regulatory cytokine interleukin-10 (IL-10), which attenuates downstream pro-inflammatory signaling [9]. Tumor necrosis factor-alpha (TNF-α), interleukin-1 beta (IL-1β), interferon-gamma (IFN-γ), and interleukin-4 (IL-4) have shown variable responses, i.e., some studies report modest elevations and others no change [10,11,12]. The overall response tends to resolve within hours to days after exercise [5,9]. The IL-6 surge that signals systemic stress also functions as a myokine that promotes muscle glycogen resynthesis, mobilizes immune cells to clear damaged tissue, and activates satellite cells involved in muscle repair and regeneration [13,14]. Neutrophil and macrophage infiltration into damaged muscle fibers both raises early muscle damage signals and enables debris clearance before remodeling [15]. The concurrent rise in IL-10 serves to constrain this response, preventing the pro-inflammatory signal from escalating into a more prolonged or dysregulated state [8]. In this sense, the inflammation is best understood as transient and adaptive, rather than lasting injury [16].

Dietary fat composition is a well-established modulator of inflammatory signaling. Saturated fatty acids (SFAs) activate Toll-Like Receptor 4 (TLR-4)-mediated Nuclear factor-κB (NF-κB) signaling, promoting transcription of pro-inflammatory cytokines including IL-6, IL-1β, and TNF-α [17,18], whereas long-chain omega-3 polyunsaturated fatty acids (PFAs)—particularly eicosapentaenoic acid (EPA) and docosahexaenoic acid (DHA)—dampen NF-κB activation, compete with arachidonic acid for cyclooxygenase and lipoxygenase enzymes, and generate specialized pro-resolving mediators that terminate inflammatory cascades [19,20]. People who habitually consume more EPA and DHA tend to have lower levels of inflammatory markers in the blood, though the strength of this relationship depends on how much omega-6 fat is consumed [21]. Monounsaturated fatty acids (MFAs) demonstrate neutral to mildly anti-inflammatory properties [22,23], while trans fatty acids promote inflammation through mechanisms overlapping with SFA [24].

Direct evidence about habitual fat quality and the exercise-induced cytokine response is still lacking. Existing research focused on short-term omega-3 supplementation trials is inconsistent [25,26]. Habitual fat intake exerts sustained effects that bolus supplementation cannot replicate: it remodels the fatty acid composition of immune cell membranes [27], calibrates eicosanoid synthesis capacity, and modulates TLR4 signaling sensitivity [28,29]. How habitual dietary fat quality modulates the post-marathon inflammatory response remains unknown. No study has examined associations between the full spectrum of habitual dietary fat subclasses and a multi-cytokine panel across pre-race, immediate post-race, and 48 hour recovery timepoints in marathon runners. Current evidence-based guidelines for endurance athletes address macronutrient quantity rather than fat subclass composition [30,31], leaving sports dietitians without actionable guidance.

This study examined associations between habitual dietary fat intake preceding a marathon and circulating IL-6, IL-10, IFN-γ, IL-1β, IL-4, and TNF-α at pre-race, immediate post-race, and 48 h post-race timepoints in recreational runners. We hypothesized that fat subclasses with established pro-inflammatory properties would be positively associated with post-race cytokine concentrations, while omega-3 PFA would demonstrate inverse associations.

## 2. Methodology

### 2.1. Participants’ Characteristics and Study Procedure

We recruited 37 marathon runners registered for a 26.2-mile marathon; 31 had habitual dietary data collected at baseline. The study protocol was previously published [32]. Briefly, participants were required to complete serum inflammatory biomarker measures during three timepoints: pre-race (~three hours before the race), immediate post-race, and 48 h post-race. Exclusion criteria were applied to the full protocol: no known cardiovascular, respiratory, gastrointestinal bleeding, inflammatory, metabolic/fluid electrolyte disorder; other chronic diseases; being pregnant; prone to vasovagal syncope because of venipuncture; or inability to attend all visits. Participants’ characteristics are listed in Table 1. The study protocol was approved by Weber State University Institutional Review Board (Subcommittee-College of Education) (approval code: IRB-AY23-24-173; Approval Date: 15 January 2024).

### 2.2. Inflammatory Biomarker Measurements

Circulating inflammatory cytokines were assessed at three timepoints: immediately before the race (pre-race), immediately post-race completion (immediate post-race), and 48 h after race completion (48 h post-race). The cytokine panel comprised IFN-γ, IL-1β, IL-4, IL-6, IL-10, and TNF-α.

Venous blood samples (10 mL) were collected from the antecubital vein into EDTA-coated plasma collection tubes. Samples were centrifuged at 1000× *g* to separate plasma, which was subsequently aliquoted and stored at −80 °C until subsequent biomarker analyses. Samples were quantified using the Luminex^®^ MAGPIX^®^ multiplexing system (Luminex Corporation, Austin, TX, USA) with a Millipore Sigma HCYTA-60K-07 MILLIPLEX^®^ Human Cytokine/Chemokine/Growth Factor Panel A configured kit for IFN-γ, IL-4, IL-6, IL-10, and TNF-α. The assay was performed according to the manufacturer’s instructions. Briefly, 50 µL of plasma was incubated with fluorescently coded magnetic beads conjugated to cytokine-specific capture antibodies, followed by incubation with biotinylated detection antibodies and streptavidin–phycoerythrin reporter. Median fluorescence intensity (MFI) was measured using the MAGPIX instrument, and cytokine concentrations were interpolated from a logistic standard curve generated from serial dilutions of a recombinant cytokine standard supplied with the kit. Samples were run in duplicate, and any sample with a coefficient of variation (CV) exceeding 20% between replicates was excluded. The lower limit of detection (LLOD) for each analyte was as specified by the manufacturer. Samples with concentrations below the LLOD were excluded from statistical analysis.

Cytokine concentrations values were log-transformed (natural logarithm) prior to statistical analysis to approximate normality and stabilize variance. The number of samples excluded due to assay quality control criteria is summarized in Table S1. Samples were excluded when biomarker concentrations were not reliably detected (ND), were below the quantification range (BLOQ), or were outside the reliable assay range (EXT). Because each participant had three collection timepoints (pre-race, post-race, and 48 h post-race), exclusion was applied at the sample/timepoint level when a value was missing or not detected.

### 2.3. Dietary Assessment

Habitual dietary fat intake over the three months preceding the race was assessed using the Diet History Questionnaire III (DHQ III), a self-administered, web-based 134-item food frequency questionnaire (FFQ) developed by the National Cancer Institute (NCI) [33,34]. Nutrient estimates were derived using the DHQ III-associated Diet Calc Analysis Software (version 1.4.3 NCI, Bethesda, MD, USA), which links reported food intakes to the USDA Food and Nutrient Database for Dietary Studies (FNDDS) [35].

We extracted 41 dietary fat variables, including total fat, solid fat, oil, dietary cholesterol, the cholesterol-saturated fat index (CSI), and the polyunsaturated-to-saturated fat ratio (PFA:SFA). Individual fatty acid subclasses comprised total and chain-specific SFA (4:0–22:0), MFA (14:1–22:1), PFA including omega-3 subclasses (ALA, EPA, DPA, DHA), trans fatty acid subclasses (trans 16:1, trans 18:1, trans 18:2), and CLA subclasses (CLA 18:2, cis9-trans11, trans10-cis12).

We energy-adjusted all dietary fat variables using the residual method [36,37] and entered them as continuous predictors in multivariable linear regression models. We additionally derived multivariable-adjusted dietary fat estimates using the residual method [36,37], further regressing each energy-adjusted fat variable on all pre-specified covariates.

All models included energy-adjusted dietary fat as a continuous predictor and were adjusted for sedentary behavior [38,39], age [40], sex [41], BMI [42,43], alcohol intake [44,45], family history of hypercholesterolemia [46,47], NSAID use [48], cholesterol medication use [49,50], smoker [51,52], and antihypertensive treatment, as each has been shown to potentially confound diet–inflammation associations (Table S2).

### 2.4. Statistical Analysis

Prior to analysis, we log-transformed all cytokine concentrations to address positive skew. We used one-way repeated measures ANOVA to examine changes in inflammatory markers across the three timepoints (pre-race, immediate post-race, and 48 h post-race). Where Mauchly’s test indicated a significant violation of sphericity, we applied Greenhouse–Geisser corrections to degrees of freedom and *p*-values. We conducted post hoc pairwise comparisons with Bonferroni correction to control for multiple comparisons and reported effect sizes as generalized eta-squared (ges). We performed all analyses in RStudio (Version 2026.01.0 + 392).

We used Spearman correlation to study multivariable-adjusted dietary fat intake and log-transformed cytokine levels. Statistical significance for all analyses was defined a priori as *p* < 0.05. Covariates being adjusted for include energy intake, sedentary status, age, biological sex, body mass index, alcohol intake, family history of hypercholesterolemia, NSAIDs, cholesterol medications, smoker, and antihypertensive treatment.

We fitted separate linear regression models for each combination of energy and multivariable-adjusted dietary fat variable and inflammatory biomarker–timepoint pair, yielding 738 models in total. All dietary fat variables were adjusted for total energy intake, sedentary behavior, age, sex, BMI, alcohol intake, family history of hypercholesterolemia, NSAID use, cholesterol medication use, smoking status, and antihypertensive treatment. To account for the inflated risk of Type I error arising from the large number of simultaneous tests, we applied the Benjamini–Hochberg false discovery rate (FDR) procedure across all 738 models [53]. We report both raw *p*-values (*p* < 0.05) and FDR-adjusted q-values (q < 0.05). Given the small sample size and the exploratory nature of the analysis, all findings should be interpreted with caution. In sensitivity analysis, we further adjusted for training volume (weekly running mileage). To assess the robustness of the 48 h MFA 20:1 associations, we conducted sensitivity analyses adding post-race NSAID use, post-race sleep deviation, and finishing time individually to the fully adjusted model. We additionally performed leave-one-out analysis, iteratively refitting the model with each participant excluded, to evaluate whether any single observation disproportionately influenced the result. Finally, we used bootstrap resampling (1000 iterations) to compare resampled confidence intervals against asymptotic estimates, given the number of covariates relative to the available sample size.

## 3. Results

### 3.1. Participant Characteristics

We included a total of 31 marathon runners (58.1% female; mean age 38.42 ± 10.15 years) with available dietary data in the analysis. Table 1 shows full participant characteristics. The participants had a healthy BMI, with mean total energy intake of 2022.97 ± 672.40 kcal/day and low alcohol consumption (1.56 ± 2.39 g/day) across the sample. Most participants were non-sedentary (93.5%). Overall, 3.2% reported a positive family history of hypercholesterolemia, 6.5% used nonsteroidal anti-inflammatory drugs (NSAIDs), 3.2% used cholesterol-lowering medication, 3.2% were current smokers, and 3.2% were receiving antihypertensive treatment. Despite their low prevalence, all covariates were retained in multivariable regression models as pre-specified confounders.

### 3.2. Habitual Dietary Fat Intake

Habitual dietary fat intake across all subclasses is presented in Table 2. After energy adjustment, total fat intake was approximately 74.6 g/day, with MFA as the largest fraction (27.84 ± 6.15 g/day), followed by SFA (24.08 ± 5.61 g/day), PFA (15.94 ± 3.33 g/day), and trans fatty acids (2.87 ± 0.99 g/day). Omega-3 and CLA intakes were low across all subclasses. Most dietary fat variables did not differ significantly between sexes. Females reported a higher PFA:SFA ratio (*p* = 0.007) and greater intakes of some short- and medium-chain SFAs and PFAs (SFA 4:0, SFA 6:0, PFA 18:3, ALA, DPA; all *p* < 0.05). Following multivariable adjustment, all between-sex differences were fully attenuated (all *p* = 1.000), as expected given that sex was included as a covariate, effectively removing sex-related variance from the dietary fat estimates used in subsequent analyses.

### 3.3. Inflammatory Marker Responses Across the Marathon Race

**Figure 1:**
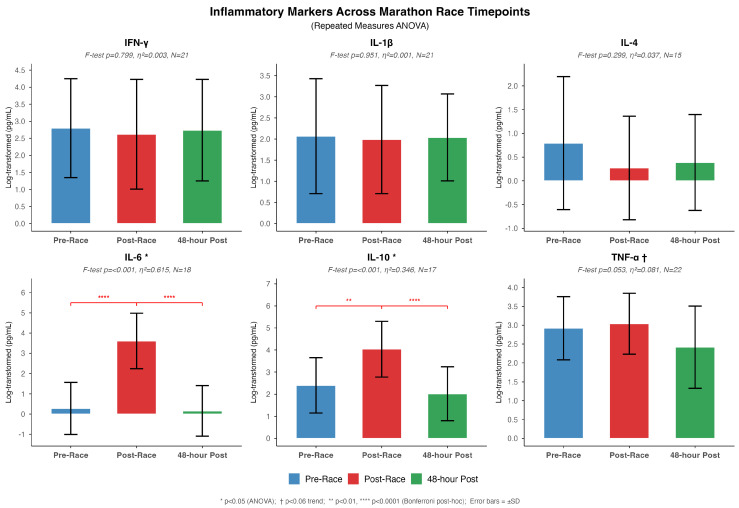
Log-transformed inflammatory marker concentrations across pre-race, immediate post-race, and 48 h post-race timepoints in marathon runners. Note: Bar graphs display mean log-transformed cytokine concentrations (± SD) at each timepoint. Sample sizes varied by biomarker due to missing data: IFN-γ (*n* = 21), IL-1β (*n* = 21), IL-4 (*n* = 15), IL-6 (*n* = 18), IL-10 (*n* = 17), and TNF-α (*n* = 22). Repeated measures ANOVA with Greenhouse–Geisser correction was applied where Mauchly’s test of sphericity was violated (IL-1β, IL-6, IL-10). Significance brackets indicate post hoc pairwise comparisons adjusted using Bonferroni correction: ** *p* < 0.01, **** *p* < 0.0001; ns = non-significant. Colors denote timepoint: blue = pre-race; red = immediate post-race; green = 48 h post-race. *η*^2^ = generalized eta squared (ges).

We presented changes in inflammatory markers across the three timepoints in Figure 1. Sample sizes varied by biomarker due to missing data (IFN-γ: *n* = 21; IL-1β: *n* = 21; IL-4: *n* = 15; IL-6: *n* = 18; IL-10: *n* = 17; TNF-α: *n* = 22). We observed significant time effects for IL-6 and IL-10, a borderline trend for TNF-α, and no significant changes for IFN-γ, IL-1β, or IL-4.

IL-6 showed the strongest response, spiking immediately post-race before returning to pre-race levels by 48 h (Greenhouse–Geisser-corrected *p* = 4.16 × 10^−8^, ges = 0.615; Mauchly’s *p* = 0.002). Post hoc comparisons confirmed a significant post-race rise and subsequent recovery (both *p* < 0.001), while pre-race and 48 h values remain statistically indistinguishable (*p* > 0.05). IL-10 followed a similar trajectory, rising significantly post-race (*p* = 0.001) before declining below pre-race levels at 48 h (post-race vs. 48 h post-race *p* < 0.001; pre-race vs. 48 h post-race *p* = 0.257; Greenhouse–Geisser-corrected *p* = 7.64 × 10^−6^, ges = 0.346; Mauchly’s *p* = 0.029). TNF-α showed a modest post-race rise followed by a decline at 48 h, but the overall time effect is nonsignificant (Greenhouse–Geisser-corrected *p* = 0.056; Mauchly’s *p* = 0.553); the post- to 48 h post-race decline approached significance before correction (raw *p* = 0.036) but did not survive Bonferroni adjustment. IFN-γ, IL-1β, and IL-4 showed no significant changes across any timepoint (all Greenhouse–Geisser-corrected *p* > 0.29).

### 3.4. Partial Correlations Between Habitual Dietary Fat Intake and Inflammatory Markers

**Figure 2:**
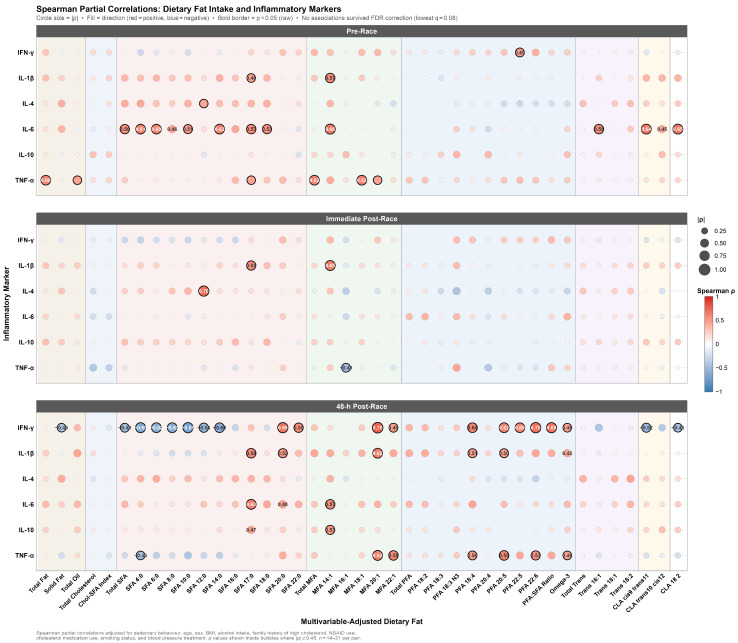
Partial Spearman correlations between multivariable-adjusted habitual dietary fat intake and log-transformed inflammatory markers across pre-race, immediate post-race, and 48 h post-race timepoints in marathon runners. Note: Each cell displays the Partial Spearman correlation coefficient (ρ) between a multivariable-adjusted dietary fat variable (x-axis) and an inflammatory marker (y-axis). Color intensity reflects the magnitude of the partial correlation: red indicates positive associations and blue indicates negative associations. Correlations were calculated between log-transformed inflammation markers and energy-adjusted dietary fatty acids further adjusting for sedentary behavior, age, sex, BMI, alcohol intake, family history of high cholesterol, NSAID use, cholesterol medication use, smoking status, and antihypertensive treatment. Inflammatory markers are log-transformed. IFN-γ = interferon-gamma; IL-1β = interleukin-1 beta; IL-4 = interleukin-4; IL-6 = interleukin-6; IL-10 = interleukin-10; TNF-α = tumor necrosis factor-alpha; SFA = saturated fatty acid; MFA = monounsaturated fatty acid; PFA = polyunsaturated fatty acid; CLA = conjugated linoleic acid; FDR = false discovery rate. ρ values shown inside bubbles are |ρ| ≥ 0.45, bold border = *p* < 0.05; no partial correlation survived FDR correction (q = 0.08).

We presented Spearman partial correlations between multivariable-adjusted dietary fat intake and log-transformed inflammatory markers across all three timepoints in Figure 2. We observed sixty nominally significant correlations; none survived false discovery rate (FDR) correction (lowest q = 0.08) (Table S3).

We summarized the correlations observed at the nominal *p* < 0.05 significance level (ρ = 0.44 to 0.66). Pre-race, total fat, total oil, total MFA, MFA 18:1 and MFA 20:1, were positively associated with TNF-α. Total SFA was positively associated with IL-6. SFA 17:0 positively associated with TNF-α, IL-6 and IL-1β. SFA subclasses (4:0,6:0,10:0,14:0, 18:0), MFA 14:1, Trans 16:1, and CLAs (cis9 trans11, 18:2) were all positively correlated to IL-6. SFA 12:0 positively associated with IL-4. MFA 14:1 was positively correlate to IL-1β. PFA 22:5 showed positive associations with IFN-γ.

Immediately post-race, we observed fewer nominally significant correlations. IL-1β correlated positively with select SFA 17:0 and MFA 14:1, IL-4 with SFA 12:0 level (ρ = 0.55 to 0.70), and TNF-α negatively with MFA 16:1 (ρ = −0.48).

At 48 h post-race, we observed the greatest number and magnitude of nominally significant correlations. Solid fat, SFAs (total, 4:0, 6:0, 8:0, 10:0, 12:0, 14:0), and CLAs (cis9 trans11, 18:2) were all negatively associated with IFN-γ (ρ = −0.69 to −0.45). SFAs (20:0, 22:0), MFAs (20:1, 22:1), PFAs (18:4, 20:5, 22:5, 22:6), PFA:SFA Ratio, and Omega-3 fatty acids all positively correlated with IFN-γ (ρ = 0.46–0.76). The dietary fats that positively correlated with IL-1β include SFAs (17:0, 20:0), MFA 20:1, and PFAs (18:4, 20:5) (ρ = 0.50–0.62). SFA 17:0 and MFA 14:1 positively correlated with IL-6. (ρ = 0.57 to 0.63). SFA (4:0) negatively correlated with TNF-α (ρ = −0.46). MFA (20:1, 22:1) and PFA (18:4, 20:5, 22:6, omega-3) positively correlated with TNF-α. (ρ = 0.46–0.63). MFA 14:1 was positively associated with IL-10.

The overall pattern of associations was largely timepoint-specific, with limited consistency across measurements.

### 3.5. Multivariable-Adjusted Associations Between Habitual Dietary Fat Intake and Inflammatory Markers

**Figure 3:**
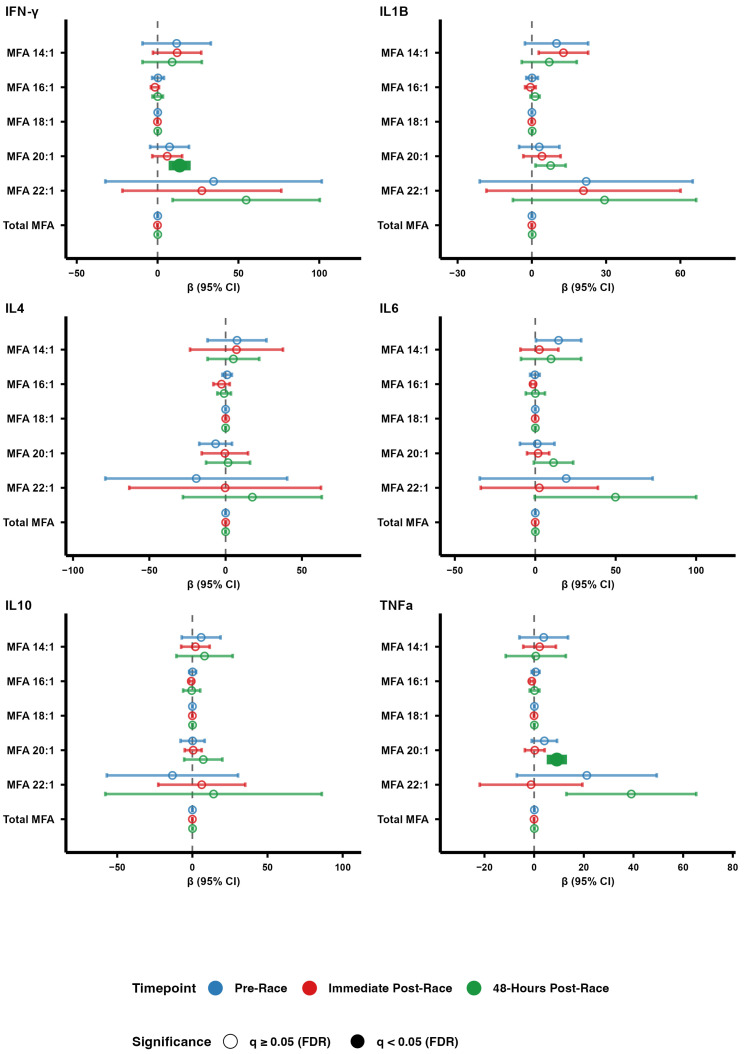
Multivariable-adjusted linear regression models between habitual MFA intake and circulating inflammatory markers at three race timepoints. Note: Each panel displays beta coefficients (β) with 95% confidence intervals from separate multivariable-adjusted linear regression models. Inflammatory biomarkers: IFN-γ = interferon-γ; IL-1β = interleukin-1β; IL-4 = interleukin-4; IL-6 = interleukin-6; IL-10 = interleukin-10; TNF-α = tumor necrosis factor-α. Timepoint colors: blue = Pre-Race; red = Immediate Post-Race; green = 48 hour Post-Race. Data points within each row are slightly offset vertically to distinguish the three timepoints. Open circles (○) and thin error bars indicate non-significant associations (q ≥ 0.05); filled circles and bold error bars indicate associations reaching statistical significance after Benjamini–Hochberg false discovery rate correction (q < 0.05). All models were adjusted for sedentary behavior, age, sex, BMI, alcohol intake, family history of high cholesterol, NSAID use, cholesterol medication use, smoking status, and high blood pressure treatment.

Multivariable-adjusted associations between habitual dietary fat intake and inflammatory markers at each timepoint are displayed in Figure 3 and Figures S1–S5. Models were adjusted for energy intake, sedentary behavior, age, sex, BMI, alcohol, family history of hypercholesterolemia, NSAID use, cholesterol medication, smoking status, and antihypertensive treatment. Effective sample sizes ranged from 18 to 21 per model due to listwise deletion (Tables S4–S9). Given the small sample size and 738 models estimated (41 fat variables × 6 biomarkers × 3 timepoints), all findings are considered exploratory.

Two associations survived Benjamini–Hochberg FDR correction (q < 0.05), both involving habitual MFA 20:1 (gondoic acid) intake at 48 h post-race: a positive association with IFN-γ (β = 13.66, 95% CI 8.94 to 18.38, q = 0.04) and a positive association with TNF-α (β = 9.14, 95% CI 6.43 to 11.86, q = 0.02). These findings are shown in Figure 3. Each 1-SD higher habitual MFA 20:1 intake (~0.10 g/day) was associated with a 3.92-fold higher 48 h post-race IFN-γ concentration (95% CI: 2.45 to 6.28, q = 0.04). A 1-SD higher habitual MFA 20:1 intake was associated with a 2.49-fold higher 48 h post-race TNF-α concentration (95% CI: 1.90 to 3.27).

Pre-race, IL-4 and IL-6 displayed nominally significant associations (*p* < 0.05) mainly with short- and medium-chain SFA subclasses. IL-4 was positively associated with solid fat, total SFA, SFA 4:0, 6:0, 10:0, 14:0, and CLA cis9 trans11, and inversely associated with the PFA:SFA ratio. IL-6 was positively associated with total SFA, SFA 4:0, 6:0, 10:0, 14:0, 18:0, MFA 14:1, Trans 16:1, CLA cis9 trans11, and CLA 18:2. IFN-γ showed a single positive association with PFA 22:5, and IL-10 with PFA 18:3 N3. IL-1β and TNF-α showed no nominally significant associations pre-race.

At immediate post-race, IL-1β was positively associated with SFA 17:0 and MFA 14:1; IL-4 with a large but imprecise negative coefficient for CLA trans10 cis12 (β = −163.31, 95% CI −310.81 to −15.81); IL-6 with PFA 18:3 N3 and Omega-3; IL-10 with SFA 8:0 and Trans 16:1; and IFN-γ with PFA 20:5 (borderline, *p* = 0.05). For TNF-α, all four nominally significant associations at this timepoint were inverse, involving total cholesterol, the cholesterol–SFA Index, MFA 16:1, and PFA 20:4.

At 48 h post-race, the broadest pattern of associations was observed, with notable directional contrasts between SFA chain lengths. For IFN-γ and TNF-α, short- and medium-chain SFA subclasses (SFA 4:0, 6:0, 8:0, 10:0, and additionally SFA 12:0 and 14:0 for TNF-α) were inversely associated, while very-long-chain SFAs (SFA 20:0, 22:0), MFA 20:1 and 22:1, several PFA subclasses (18:4, 20:5, 22:5, 22:6), and the PFA:SFA ratio were positively associated. The MFA 20:1 association for both IFN-γ and TNF-α survived FDR correction. MFA 20:1 was positively associated with IFN-γ concentrations at 48 h post-race (β = 13.66; 95% CI: 8.94, 18.38, *p* < 0.01, q = 0.04) and TNF-α (β = 9.14; 95% CI: 6.43, 11.86, *p* < 0.01, q = 0.02). IL-1β was positively associated with total fat, SFA 16:0, SFA 17:0, MFA 18:1, and MFA 20:1. IL-4 was positively associated with SFA 4:0, 6:0, and CLA cis9 trans11, and inversely with PFA 20:4. IL-6 was positively associated with total SFA, SFA 14:0, and SFA 20:0. IL-10 showed a single strong positive association with the PFA:SFA ratio (β = 0.23, 95% CI 0.12 to 0.34, *p* < 0.01).

Across all timepoints, confidence intervals were generally wide, reflecting the limited sample size. Apart from the two MFA 20:1 associations at 48 h post-race, no other associations survived FDR correction, and the remaining patterns of nominally significant associations were largely timepoint-specific, with no dietary fat variable showing consistent directionality across all three measurement points.

### 3.6. Tables and Figures

This study examined associations between habitual dietary fat intake and the acute cytokine response at pre-race, immediate post-race, and 48 h post-race timepoints in recreational marathon runners. Repeated measures ANOVA confirmed a robust multi-cytokine inflammatory response: IL-6 and IL-10 demonstrated large-magnitude significant elevations across timepoints, with TNF-α showing a borderline trend. We found that MFA 20:1 was positively associated with IFN-γ and TNF-α at 48 h post-race. This remained significant after Benjamini–Hochberg FDR correction across 738 regression models.

The remaining pattern of nominally significant associations, while not surviving FDR correction, was timepoint-specific. Pre-race associations centered on short- and medium-chain SFA subclasses with IL-4 and IL-6. Immediate post-race associations were sparse and largely involved cholesterol-related variables. Finally, 48 h post-race associations were the broadest, with inverse associations for short- and medium-chain SFAs and one long-chain SFA, and a coherent positive cluster involving long-chain MFAs and PFAs. This timepoint-specific signature suggests that the 48 h post-race period was the window in which habitual diet most clearly modulates circulating cytokines.

## 4. Discussion

### 4.1. Marathon-Induced Inflammatory Response

The cytokine pattern observed aligns with established exercise immunology. IL-6 rose markedly post-race, consistent with its role as the primary exercise-associated myokine, released in proportion to exercise duration, active muscle mass, and glycogen depletion [12,54,55,56,57,58]. The concurrent IL-10 elevation reflects the counter-regulatory cascade driven by IL-6: IL-6 stimulates IL-10 secretion, which attenuates downstream NF-κB-mediated pro-inflammatory transcription [9,12,57]. The borderline TNF-α response and muted IFN-γ, IL-1β, and IL-4 responses are consistent with prior reports describing variable responses of these cytokines to endurance running, attributable to inter-individual differences in training status, race conditions, and nutritional state [12,25,59,60,61].

### 4.2. Eicosenoic Acid (MFA 20:1) Associations with Inflammation at 48 Hours Post-Race

It should be noted that unlike IL-6 and IL-10, IFN-γ and TNF-α did not demonstrate significant temporal changes across the three timepoints (Figure 1). The associations reported here therefore reflect habitual dietary fat intake in relation to 48 h post-race cytokine concentrations rather than a directional inflammatory resolution trajectory and should be interpreted accordingly.

Eicosenoic acid is found in plant oils, nuts, jojoba oil, and marine oils [62,63,64]. To the best of our knowledge, no paper directly examines 20:1 MFA intake vs. IFN-γ/TNF-α in athletes. MUFA-focused human and animal data suggest neutral or anti-inflammatory effects (lower TNF-α/IFN-γ) [65,66]. However, existing reviews and experimental work describe oleic acid (18:1) as the main MFA in human diet and circulation, central to Mediterranean and olive oil research [67,68,69,70]. Further, MFA effects can be dose-dependent and non-linear. An in vitro human monocyte study shows that MFAs can be anti-inflammatory at intermediate concentrations while pro-inflammatory at very high or low doses [71]. The dominant anti-inflammatory role of MFA has been built largely from oleic acid in resting populations, while distinct long-chain MFAs, host states, and intake ranges remain uncharacterized. The finding emerged exclusively at the 48 h post-race timepoint, consistent with our broader pattern of timepoint-specific associations and supporting the interpretation that habitual fat quality–cytokine association may be most detectable after acute exercise-induced signaling has subsided. The available literature in endurance athletes and runners largely tests omega-3 PFAs, not MFAs, for effects on post-exercise cytokines. However, they do show modulation of TNF-α and IL-6 with heterogeneous and time-dependent patterns [72,73]. In summary, mechanistic work indicates that immune effects of MFAs are molecule-specific, non-linear, and context-dependent. This provides a plausible framework where a long-chain MFA such as 20:1 is positively associated with IFN-γ and TNF-α during the 48 h post-marathon resolution phase.

### 4.3. SFAs, PFAs Associations with Inflammation Markers at 48 Hours Post-Race

In both the 48 h post-race correlation and multivariable linear regression studies we found that short- and medium-chain chain SFAs and one long-chain SFA were nominally inversely associated with IFN-gamma and TNF alpha. The finding is considered exploratory as it did not pass FDR-adjustment. This has been supported by the current literature. Butyrate (SFA 4:0) has been reported to show anti-inflammatory effects on leukocytes and endothelial cells by reducing TNF-alpha production via free fatty acid receptors 2/3 (G protein-coupled receptors) and histone deacetylase inhibition [74,75,76]. Direct data on SFA6:0 (caproic acid) and IFN-γ/TNF-α in vivo are lacking, and it shows a partial, atypical anti-inflammatory pattern in hepatic cells (downregulates NF-κB, IL-8 unchanged) [77]; mechanistic and intervention studies are needed. Multiple in vivo and in vitro studies show SFA8:0 (caprylic) dampens NF-κB-driven cytokines (TNF-α, IFN-γ-related pathways) via TLR4/NF-κB suppression, ABCA1–JAK2–STAT3 activation, and reduced oxidative stress [78,79,80,81,82,83]. Lauric acid (SFA12:0) exhibits bidirectional, context-dependent immunomodulation. In naïve epithelial and dendritic cells, it can activate NF-κB and pro-inflammatory pathways [84,85,86], but in multiple models of established inflammation, it reduces NF-κB activation and pro-inflammatory cytokines or adhesion molecules, including TNF-α, IL-6, IL-1β, ICAM-1 and VCAM-1 [87,88,89,90,91]. The observed inverse associations between circulating SFA12:0 and IFN-γ/TNF-α at 48 h post-marathon may therefore reflect a pro-resolving role of lauric acid under recovery conditions, rather than a uniformly pro- or anti-inflammatory effect. Myristic acid (SFA 14:0) is a long-chain SFA, but current data show clear in vitro anti-inflammatory activity by increased IL-10 in LPS-stimulated macrophages and systemic anti-inflammatory effect in vivo [92]. In BV-2 microglial cells, co-treatment with myristic and heptadecanoic acids downregulated IL-1β, IL-6, and TNF-α and inhibited LPS-induced inflammation via the NF-κB pathway [93]. Myristic acid specifically limit IFN and related interferon signaling via attenuated cGAS–STING-induced interferon responses [94]. Mechanistic and in vivo studies support that several short- and medium-chain SFAs (4:0, 6:0, 8:0, 12:0) and myristic acid (14:0) can exert context-dependent anti-inflammatory and pro-resolving actions—consistent with their inverse associations with IFN-γ and TNF-α 48 h post-marathon.

At 48 h post-race, long-chain PFA subclasses (18:4, 20:5, 22:5, 22:6) and the PFA:SFA ratio were positively associated with IFN-γ and TNF-α. The finding appears to contradict the established anti-inflammatory profile of omega-3 fatty acids [20]. However, the literature is built primarily on supplementation trials in resting populations or chronic inflammatory disease cohorts, where EPA/DHA reduce baseline TNF-α and IL-6 [95]. Several exercise-induced muscle damage randomized clinical trials report no significant difference in IL-6 or TNF-α between omega-3 and placebo [26,96]. The translation of the anti-inflammatory PUFA effect into a recovery-phase exercise context is therefore not assured.

### 4.4. Strengths and Limitations

To our knowledge, the study is the first to evaluate a broad dietary fat panel against a six-cytokine panel across three marathon-specific timepoints. We controlled the false discovery rate rigorously and transparently. All habitual dietary fat intake variables were energy-adjusted, thereby isolating dietary fat composition from absolute intake.

The primary limitation was a modest sample size, which constrained power to detect small-to-moderate associations after multiple variable correction. Estimates from the fully adjusted models should be interpreted with appropriate caution given the number of covariates relative to sample size. Bootstrap resampling of the primary models produced wider confidence intervals than the asymptotic estimates (IFN-γ: 2.61 to 27.61 vs. 8.94 to 18.38; TNF-α: 2.27 to 13.74 vs. 6.43 to 11.86), indicating the standard errors from the fully adjusted models likely understate true sampling uncertainty. However, both resampled intervals remained entirely positive. The association between habitual MFA 20:1 intake and post-race inflammatory markers also remained directionally consistent and statistically significant across a series of progressively simpler model specifications with fewer covariates (Table S10); leave-one-out analysis showed no single participant disproportionately drove the result, suggesting the underlying association is unlikely to be solely a product of model overfitting.

Training volume (weekly mileage) was collected but not included in models, as the available degrees of freedom per model precluded adjustment beyond the pre-specified covariate set. However, we addressed two of the three principal causal pathways through which training volume could plausibly confound diet–cytokine associations. We adjusted for energy intake, capturing variation in caloric consumption driven by training-related energy demands, and we adjusted for sedentary behavior, distinguishing active from inactive participants. The remaining pathway—direct training-induced modulation of innate immune signaling [97,98]—is not fully captured by proxy. In sensitivity analyses further adjusting for weekly running mileage, associations were attenuated but remained directionally consistent and statistically significant (Table S11). These findings suggest that the observed diet–cytokine associations were not materially explained by training volume. For alcohol, although a dedicated recovery period log was not administered, 24 h dietary recall data at 48 h post-race collected using the validated Automated Self-Administered 24 h Dietary Assessment Tool (ASA24) [99] were available. In sensitivity analyses, we individually incorporated post-race NSAID use, post-race sleep deviation, finish time, or recovery period alcohol intake, associations remained consistent and statistically significant (Table S11). We did not systematically record exercise activity during the 48 h recovery window. Residual confounding therefore cannot be excluded and represents a priority for future work with larger samples.

Self-reported dietary intake may be subject to recall bias and measurement error, though we used a validated food frequency questionnaire with moderate-to-good agreement against 24 h recalls for total fat and fat subclasses [100,101]. Agreement between DHQ II and DHQ III versions has been confirmed in an independent cohort [102]. Analysis of acute peri-race nutrition was beyond the scope of the present study and represents a direction for future work.

## 5. Conclusions

Habitual intake of the long-chain monounsaturated fatty acid 20:1 appears to be positively associated with IFN-γ and TNF-α at 48 h post-race. This finding emerged specifically at the 48 h post-race timepoint rather than the acute response phase. This suggests that the 48 h post-race timepoint, once acute exercise-induced signaling has subsided, may be a window in which habitual dietary fat quality is most detectable in relation to cytokine concentrations. Given the exploratory nature and modest sample size of this study, these findings should be interpreted as hypothesis-generating rather than conclusive and require replication in larger cohorts before informing dietary recommendations for endurance athletes.

## Figures and Tables

**Table 1:** Participant characteristics of marathon runners.

Characteristics	Total (*n* = 31, 100%)	Male (*n* = 13, 41.9%)	Female (*n* = 18, 58.1%)
Age (years)	38.42 ± 10.15	39.15 ± 11.49	37.89 ± 9.39
BMI (kg/m^2^)	23.74 ± 3.67	24.78 ± 2.96	22.92 ± 4.06
Total energy intake (kcal)	2022.97 ± 672.40	2155.11 ± 536.86	1927.54 ± 755.92
Sedentary, *n* (%)	2 (6.5)	0 (0.0)	2 (100.0)
Alcohol (g)	1.56 ± 2.39	1.81 ± 2.76	1.39 ± 2.14
Family history of hypercholesterolemia, *n* (%)			
Yes	1 (3.2)	1 (100.0)	0 (0.0)
No	17 (54.8)	8 (47.1)	9 (52.9)
Don’t know	9 (29.0)	3 (33.3)	6 (66.7)
NSAID use, *n* (%)	2 (6.5)	1 (50.0)	1 (50.0)
Cholesterol medication use, *n* (%)	1 (3.2)	1 (100.0)	0 (0.0)
Current smoker, *n* (%)	1 (3.2)	0 (0.0)	1 (100.0)
Antihypertensive treatment, *n* (%)	1 (3.2)	1 (100.0)	0 (0.0)

Note. Values for continuous variables are reported as mean ± SD. Categorical variables are reported as *n* (column %). BMI = body mass index; NSAID = nonsteroidal anti-inflammatory drug.

**Table 2:** Habitual dietary fat intake of marathon runners (N = 31).

Dietary Fat Variable	Energy-Adjusted (Mean ± SD)				Multivariable-Adjusted (Mean ± SD)			
	Total (N = 31)	Male (N = 13)	Female (N = 18)	*p*-Value	Total (N = 31)	Male (N = 13)	Female (N = 18)	*p*-Value
Total fat (g/day)	74.61 ± 11.80	72.04 ± 10.68	76.47 ± 12.50	0.298	74.61 ± 10.09	74.61 ± 8.36	74.61 ± 11.58	1.000
Solid fat (g/day)	29.54 ± 9.26	28.05 ± 6.77	30.62 ± 10.77	0.423	32.53 ± 8.74	32.53 ± 8.21	32.53 ± 9.42	1.000
Oil (g/day)	24.70 ± 8.80	22.89 ± 7.33	26.01 ± 9.71	0.317	24.70 ± 6.28	24.70 ± 6.36	24.70 ± 6.43	1.000
Cholesterol (mg/day)	302.77 ± 150.16	312.45 ± 181.41	295.77 ± 128.20	0.779	302.77 ± 119.41	302.77 ± 145.63	302.77 ± 99.08	1.000
Cholesterol–SFA Index	39.86 ± 9.71	39.39 ± 9.13	40.19 ± 10.35	0.821	39.86 ± 8.15	39.86 ± 7.42	39.86 ± 8.95	1.000
PFA:SFA Ratio	24.55 ± 13.22	17.93 ± 4.86	29.33 ± 15.30	**0.007**	24.55 ± 11.85	24.55 ± 5.43	24.55 ± 15.42	1.000
**Saturated Fatty Acids (SFAs)**								
Total SFA (g/day)	24.08 ± 5.61	22.80 ± 4.09	25.00 ± 6.44	0.256	24.08 ± 4.87	24.08 ± 4.42	24.08 ± 5.35	1.000
SFA 4:0 Butanoic (g/day)	0.55 ± 0.31	0.40 ± 0.23	0.66 ± 0.32	**0.012**	0.55 ± 0.23	0.55 ± 0.23	0.55 ± 0.24	1.000
SFA 6:0 Hexanoic (g/day)	0.32 ± 0.21	0.23 ± 0.15	0.39 ± 0.23	**0.022**	0.32 ± 0.16	0.32 ± 0.17	0.32 ± 0.16	1.000
SFA 8:0 Octanoic (g/day)	0.29 ± 0.21	0.24 ± 0.23	0.32 ± 0.18	0.323	0.29 ± 0.19	0.29 ± 0.24	0.29 ± 0.15	1.000
SFA 10:0 Decanoic (g/day)	0.52 ± 0.26	0.43 ± 0.24	0.59 ± 0.26	0.102	0.52 ± 0.23	0.52 ± 0.26	0.52 ± 0.21	1.000
SFA 12:0 Dodecanoic (g/day)	0.95 ± 0.84	1.11 ± 1.25	0.84 ± 0.33	0.462	0.95 ± 0.82	0.95 ± 1.10	0.95 ± 0.55	1.000
SFA 14:0 Tetradecanoic (g/day)	2.19 ± 1.02	1.87 ± 0.81	2.42 ± 1.11	0.118	2.19 ± 0.87	2.19 ± 0.94	2.19 ± 0.84	1.000
SFA 16:0 Hexadecanoic (g/day)	12.74 ± 2.53	12.09 ± 1.83	13.21 ± 2.89	0.196	12.74 ± 2.02	12.74 ± 1.41	12.74 ± 2.45	1.000
SFA 17:0 Margaric (g/day)	0.11 ± 0.04	0.11 ± 0.04	0.12 ± 0.05	0.884	0.11 ± 0.04	0.11 ± 0.03	0.11 ± 0.04	1.000
SFA 18:0 Octadecanoic (g/day)	5.42 ± 1.27	5.26 ± 0.92	5.53 ± 1.49	0.540	5.42 ± 1.02	5.42 ± 0.88	5.42 ± 1.15	1.000
SFA 20:0 Arachidic (g/day)	0.17 ± 0.06	0.17 ± 0.05	0.17 ± 0.07	0.991	0.17 ± 0.05	0.17 ± 0.06	0.17 ± 0.05	1.000
SFA 22:0 Behenic (g/day)	0.21 ± 0.13	0.21 ± 0.12	0.21 ± 0.14	0.989	0.21 ± 0.11	0.21 ± 0.12	0.21 ± 0.11	1.000
**Monounsaturated Fatty Acids (MFAs)**								
Total MFA (g/day)	27.84 ± 6.15	26.90 ± 5.11	28.51 ± 6.87	0.461	27.84 ± 5.15	27.84 ± 4.37	27.84 ± 5.85	1.000
MFA 14:1 Myristoleic (g/day)	0.12 ± 0.07	0.13 ± 0.06	0.12 ± 0.07	0.940	0.12 ± 0.05	0.12 ± 0.05	0.12 ± 0.06	1.000
MFA 16:1 Hexadecenoic (g/day)	0.99 ± 0.35	0.99 ± 0.31	0.99 ± 0.39	0.957	0.99 ± 0.33	0.99 ± 0.25	0.99 ± 0.39	1.000
MFA 18:1 Octadecenoic (g/day)	25.89 ± 5.87	24.92 ± 4.80	26.58 ± 6.58	0.423	25.89 ± 4.76	25.89 ± 4.08	25.89 ± 5.39	1.000
MFA 20:1 Eicosenoic (g/day)	0.27 ± 0.10	0.30 ± 0.12	0.25 ± 0.08	0.192	0.27 ± 0.08	0.27 ± 0.10	0.27 ± 0.06	1.000
MFA 22:1 Docosenoic (g/day)	0.01 ± 0.02	0.02 ± 0.02	0.01 ± 0.02	0.206	0.01 ± 0.02	0.01 ± 0.02	0.01 ± 0.01	1.000
**Polyunsaturated Fatty Acids (PFAs)**								
Total PFA (g/day)	15.94 ± 3.33	15.71 ± 3.03	16.10 ± 3.60	0.742	15.94 ± 2.40	15.94 ± 2.70	15.94 ± 2.23	1.000
PFA 18:2 Octadecadienoic (g/day)	13.99 ± 3.17	13.83 ± 2.84	14.11 ± 3.47	0.808	13.99 ± 2.23	13.99 ± 2.48	13.99 ± 2.10	1.000
PFA 18:3 Octadecatrienoic (g/day)	1.40 ± 0.35	1.23 ± 0.31	1.52 ± 0.34	**0.018**	1.40 ± 0.30	1.40 ± 0.29	1.40 ± 0.32	1.000
PFA 18:3 N3 Alpha-linolenic (g/day)	1.31 ± 0.39	1.16 ± 0.32	1.43 ± 0.41	**0.049**	1.31 ± 0.34	1.31 ± 0.26	1.31 ± 0.40	1.000
PFA 18:4 Octadecatetraenoic (g/day)	0.01 ± 0.02	0.02 ± 0.02	0.01 ± 0.01	0.106	0.01 ± 0.01	0.01 ± 0.01	0.01 ± 0.01	1.000
PFA 20:4 Eicosatetraenoic (g/day)	0.14 ± 0.09	0.16 ± 0.12	0.13 ± 0.07	0.336	0.14 ± 0.07	0.14 ± 0.10	0.14 ± 0.05	1.000
PFA 20:5 EPA (g/day)	0.04 ± 0.05	0.05 ± 0.07	0.02 ± 0.03	0.164	0.04 ± 0.04	0.04 ± 0.05	0.04 ± 0.03	1.000
PFA 22:5 DPA (g/day)	0.02 ± 0.02	0.03 ± 0.02	0.01 ± 0.01	**0.029**	0.02 ± 0.01	0.02 ± 0.02	0.02 ± 0.01	1.000
PFA 22:6 DHA (g/day)	0.08 ± 0.08	0.11 ± 0.10	0.06 ± 0.05	0.200	0.08 ± 0.06	0.08 ± 0.07	0.08 ± 0.04	1.000
Omega-3 (g/day)	1.51 ± 0.43	1.43 ± 0.45	1.57 ± 0.42	0.398	1.51 ± 0.36	1.51 ± 0.34	1.51 ± 0.38	1.000
**Trans Fatty Acids**								
Total Trans FA (g/day)	2.87 ± 0.99	3.10 ± 0.69	2.70 ± 1.14	0.230	2.87 ± 0.75	2.87 ± 0.78	2.87 ± 0.76	1.000
Trans 16:1 (g/day)	0.05 ± 0.03	0.04 ± 0.02	0.06 ± 0.03	0.078	0.05 ± 0.02	0.05 ± 0.02	0.05 ± 0.02	1.000
Trans 18:1 Elaidic (g/day)	2.43 ± 0.89	2.69 ± 0.62	2.24 ± 1.02	0.137	2.43 ± 0.66	2.43 ± 0.70	2.43 ± 0.66	1.000
Trans 18:2 Linolelaidic (g/day)	0.35 ± 0.10	0.35 ± 0.08	0.35 ± 0.12	0.828	0.35 ± 0.09	0.35 ± 0.10	0.35 ± 0.09	1.000
**Conjugated Linoleic Acid (CLA)**								
CLA 18:2 Linoleic (g/day)	0.11 ± 0.06	0.10 ± 0.05	0.12 ± 0.06	0.221	0.11 ± 0.04	0.11 ± 0.04	0.11 ± 0.05	1.000
CLA cis9-trans11 (g/day)	0.10 ± 0.05	0.08 ± 0.04	0.10 ± 0.05	0.221	0.10 ± 0.04	0.10 ± 0.04	0.10 ± 0.04	1.000
CLA trans10-cis12 (g/day)	0.02 ± 0.01	0.02 ± 0.01	0.02 ± 0.01	0.981	0.02 ± 0.01	0.02 ± 0.01	0.02 ± 0.01	1.000

Note. Values are mean ± SD. Bold *p*-values indicate statistical significance (*p* < 0.05, Welch’s *t*-test by sex). Energy-adjusted values derived from residual method. Multivariable-adjusted values additionally adjusted for sedentary behavior, age, sex, BMI, alcohol intake, family history of high cholesterol, NSAID use, cholesterol medication use, smoking status, and blood pressure treatment.

## Data Availability

Further inquiries can be directed to the corresponding author.

## Supplementary Materials

The following supporting information can be downloaded at: https://www.mdpi.com/article/10.3390/nu18142273/s1, Table S1. Number of samples excluded based on assay detection limits by biomarker and timepoint. Table S2. Description of the covariates used in the current study. Table S3. Nominally significant Spearman partial correlations between dietary fat intake and circulating inflammatory markers in marathon runners across three race timepoints. Figure S1. Multivariable-adjusted linear regression models between habitual overall fat intake and circulating inflammatory markers at three race timepoints. Figure S2. Multivariable-adjusted linear regression models between habitual cholesterol-related intake and circulating inflammatory markers at three race timepoints. Figure S3. Multivariable-adjusted linear regression models between habitual SFA-related intake and circulating inflammatory markers at three race timepoints. Figure S4. Multivariable-adjusted linear regression models between habitual PFA-related intake and circulating inflammatory markers at three race timepoints. Figure S5. Multivariable-adjusted linear regression models between habitual TRANs fat intake and circulating inflammatory markers at three race timepoints. Table S4. Multivariable-adjusted linear regression associations between habitual dietary fat intake and circulating IFN-γ at three race-related timepoints. Table S5. Multivariable-adjusted linear regression associations between habitual dietary fat intake and circulating Interleukin-1β (IL-1β) at three race-related timepoints. Table S6. Multivariable-adjusted linear regression associations between habitual dietary fat intake and circulating Interleukin-4 (IL-4) at three race-related timepoints. Table S7. Multivariable-adjusted linear regression associations between habitual dietary fat intake and circulating Interleukin-6 (IL-6) at three race-related timepoints. Table S8. Multivariable-adjusted linear regression associations between habitual dietary fat intake and circulating Interleukin-10 (IL-10) at three race-related timepoints. Table S9. Multivariable-adjusted linear regression associations between habitual dietary fat intake and circulating Tumor Necrosis Factor-α (TNF-α) at three race-related timepoints. Table S10. Sensitivity of the association between habitual MFA 20:1 (eicosenoic acid) intake and 48 h post-race inflammatory markers across progressively adjusted model specifications, with leave-one-out and bootstrap stability checks on the fully adjusted model. Table S11. Association of Energy- and Covariate-Adjusted MFA 20:1 (Eicosenoic Acid) Intake with Post-Race Inflammatory Cytokines: Primary Models and Sensitivity Analysis Further Adjusting for Training Volume. Table S12. Sensitivity analysis of the association between habitual MFA 20:1 intake and 48 h post-race IFN-γ and TNF-α, additionally adjusting for post-race recovery-period behaviors.
