# Supplementary material for: The Associations Between Habitual Dietary Fat Intake and Inflammatory Markers Among Marathon Runners: An Exploratory Study
Source: Nutrients. 2026 Jul 11;18(14):2273. doi: 10.3390/nu18142273 (PMC13414860; doi:10.3390/nu18142273)
Supplement: Supplementary file 1 [file nutrients-18-02273-s001.zip › nutrients-4401228-supplementary.pdf]

Supplementary Tables and Figures

Table S1. Number of samples excluded based on assay detection limits by biomarker and time point

Biomarker	Time point	Samples analyzed (n)	Samples excluded (n)	Reason for exclusion
IFN-γ	Pre	25	1	Not detected / below assay detection limit
IFN-γ	Post	25	1	Not detected / below assay detection limit
IFN-γ	48 h (F)	24	0	—
IL-1β	Pre	25	1	Not detected / below assay detection limit
IL-1β	Post	25	0	—
IL-1β	48 h (F)	24	1	Not detected / below assay detection limit
IL-4	Pre	25	2	Not detected / below assay detection limit
IL-4	Post	25	7	Not detected / below assay detection limit
IL-4	48 h (F)	24	7	Not detected / below assay detection limit
IL-6	Pre	25	3	Not detected / below assay detection limit
IL-6	Post	25	1	Not detected / below assay detection limit
IL-6	48 h (F)	24	4	Not detected / below assay detection limit
IL-10	Pre	25	3	Not detected / below assay detection limit

IL-10	Post	25	1	Not detected / below assay detection limit
IL-10	48 h (F)	24	2	Not detected / below assay detection limit
TNF-α	Pre	25	1	Not detected / below assay detection limit
TNF-α	Post	25	1	Not detected / below assay detection limit
TNF-α	48 h (F)	24	0	—

Table S2. Description of the covariates used in the current study

Covariates	Variable Description
Total energy intake	Dietary energy intake (kcal/day)
BMI	Continuous, kg/m^2
Sedentary behavior	Would you characterize your lifestyle as "sedentary"? Yes/no
Age	Age on the Ogden Marathon race day (May 18, 2024), Years
Sex	Biological Sex: 1= male ; 2 = female
Alcohol intake (habitual)	g/day, collected using DHQ III at baseline
Family history of hypercholesterolemia	Have your mother, father, or siblings suffered from (please select all that apply): High cholesterol

	Yes/no
NSAID use	Yes/no, 1 = NSAIDS/Anti-inflammatories (Motrin, Advil)
Cholesterol medication use	Please Select Any Medications You Are Currently Using: Cholesterol<br>1 = Cholesterol
Smoker	Are you a cigarette smoker?<br>(Yes/No)
Antihypertensive treatment	Are you currently being treated for high blood pressure?<br>Yes/No
Covariates used in sensitivity analysis	
Training volume/Weekly milage	Continuous. During your typical training week not specific to the current marathon training, how many miles would you run (per week)? Please enter your average weekly miles (ex. 15, if you run 15 miles per week).
NSAID use after race	Have you taken NSAIDs since finishing the race?<br>Yes/no
Sleep deviation	Sleep deviation the night following the marathon, assessed via a follow-up questionnaire administered at the 48-hour visit<br><br>Compared to your typical night of sleep, was this amount (from night after race)_______?<br>1 = below average, 2 = average, 3 = more than average
Recovery-period alcohol intake	measured using 24-hour dietary recalls collected at 48-hour post-race collected using the validated Automated Self-Administered 24 h Dietary Assessment Tool (ASA24).[50]
Finish line time	Ogden Marathon (2024) finish time in minutes

Table S3. Nominally significant Spearman partial correlations between dietary fat intake and circulating inflammatory markers in marathon runners across three race timepoints.

Timepoint	Inflammatory Marker	Dietary Fat	ρ	p (raw)	q (FDR)
Pre-Race					
	IFN-γ	PFA 22:5	0.49	0.028	0.467
	IL-1β	SFA 17:0	0.46	0.037	0.536
	IL-1β	MFA 14:1	0.57	0.007	0.193
	IL-4	SFA 12:0	0.44	0.050	0.625
	IL-6	Total SFA	0.56	0.016	0.328
	IL-6	SFA 4:0	0.61	0.008	0.216
	IL-6	SFA 6:0	0.60	0.008	0.222
	IL-6	SFA 10:0	0.51	0.029	0.467
	IL-6	SFA 14:0	0.62	0.006	0.176
	IL-6	SFA 17:0	0.57	0.013	0.280
	IL-6	SFA 18:0	0.53	0.023	0.418
	IL-6	MFA 14:1	0.66	0.003	0.176
	IL-6	Trans 16:1	0.58	0.011	0.271
	IL-6	CLA cis9 trans11	0.64	0.004	0.176
	IL-6	CLA 18:2	0.65	0.004	0.176
	TNF-α	Total Fat	0.60	0.005	0.176
	TNF-α	Total Oil	0.45	0.049	0.625
	TNF-α	SFA 17:0	0.45	0.049	0.625
	TNF-α	Total MFA	0.62	0.004	0.176
	TNF-α	MFA 18:1	0.62	0.003	0.176

Timepoint	Inflammatory Marker	Dietary Fat	ρ	p (raw)	q (FDR)
	TNF-α	MFA 20:1	0.45	0.049	0.625
Immediate Post-Race					
	IL-1β	SFA 17:0	0.55	0.012	0.280
	IL-1β	MFA 14:1	0.65	0.002	0.176
	IL-4	SFA 12:0	0.70	0.004	0.176
	TNF-α	MFA 16:1	−0.48	0.038	0.542
48-h Post-Race					
	IFN-γ	Solid Fat	−0.45	0.046	0.624
	IFN-γ	Total SFA	−0.48	0.032	0.487
	IFN-γ	SFA 4:0	−0.65	0.002	0.176
	IFN-γ	SFA 6:0	−0.62	0.004	0.176
	IFN-γ	SFA 8:0	−0.63	0.003	0.176
	IFN-γ	SFA 10:0	−0.69	7.21×10^{-4}	0.140
	IFN-γ	SFA 12:0	−0.54	0.015	0.306
	IFN-γ	SFA 14:0	−0.60	0.005	0.176
	IFN-γ	SFA 20:0	0.69	7.41×10^{-4}	0.140
	IFN-γ	SFA 22:0	0.55	0.011	0.271
	IFN-γ	MFA 20:1	0.76	1.09×10^{-4}	0.083
	IFN-γ	MFA 22:1	0.49	0.029	0.467
	IFN-γ	PFA 18:4	0.60	0.005	0.176
	IFN-γ	PFA 20:5	0.62	0.003	0.176
	IFN-γ	PFA 22:5	0.60	0.005	0.176

Timepoint	Inflammatory Marker	Dietary Fat	ρ	p (raw)	q (FDR)
	IFN-γ	PFA 22:6	0.70	5.28×10^{-4}	0.140
	IFN-γ	PFA:SFA Ratio	0.68	0.001	0.155
	IFN-γ	Omega-3	0.46	0.040	0.554
	IFN-γ	CLA cis9 trans11	−0.52	0.020	0.390
	IFN-γ	CLA 18:2	−0.46	0.040	0.554
	IL-1β	SFA 17:0	0.58	0.009	0.229
	IL-1β	SFA 20:0	0.52	0.022	0.407
	IL-1β	MFA 20:1	0.62	0.004	0.176
	IL-1β	PFA 18:4	0.51	0.027	0.467
	IL-1β	PFA 20:5	0.50	0.029	0.467
	IL-6	SFA 17:0	0.63	0.009	0.222
	IL-6	MFA 14:1	0.57	0.020	0.397
	IL-10	MFA 14:1	0.53	0.030	0.467
	TNF-α	SFA 4:0	−0.46	0.037	0.536
	TNF-α	MFA 20:1	0.63	0.002	0.176
	TNF-α	MFA 22:1	0.58	0.006	0.177
	TNF-α	PFA 18:4	0.50	0.022	0.408
	TNF-α	PFA 20:5	0.59	0.005	0.176
	TNF-α	PFA 22:6	0.53	0.013	0.288
	TNF-α	Omega-3	0.46	0.035	0.535

ρ, Spearman partial correlation coefficient; **p (raw)**, unadjusted p-value; **q (FDR)**, Benjamini–Hochberg false discovery rate–adjusted p-value. All associations are nominally significant ($p < 0.05$); none survived FDR correction (lowest $q = 0.083$). Negative associations are indicated with a minus sign (−).

Partial correlation covariates: sedentary behaviour, age, sex, BMI, alcohol intake, family history of high cholesterol, NSAID use, cholesterol medication use, smoking status, and blood pressure treatment. n = 14–21 per pair.

IFN-γ, interferon-γ; **IL-1β**, interleukin-1β; **IL-4**, interleukin-4; **IL-6**, interleukin-6; **IL-10**, interleukin-10; **TNF-α**, tumour necrosis factor-α.

SFA, saturated fatty acid; **MFA**, monounsaturated fatty acid; **PFA**, polyunsaturated fatty acid; **CLA**, conjugated linoleic acid. Carbon chain notation: chain length:number of double bonds (e.g., SFA 17:0 = margaric acid; MFA 14:1 = myristoleic acid; MFA 16:1 = palmitoleic acid; MFA 20:1 = gondoic acid; MFA 22:1 = erucic acid; PFA 18:4 = stearidonic acid; PFA 22:6 = docosahexaenoic acid).

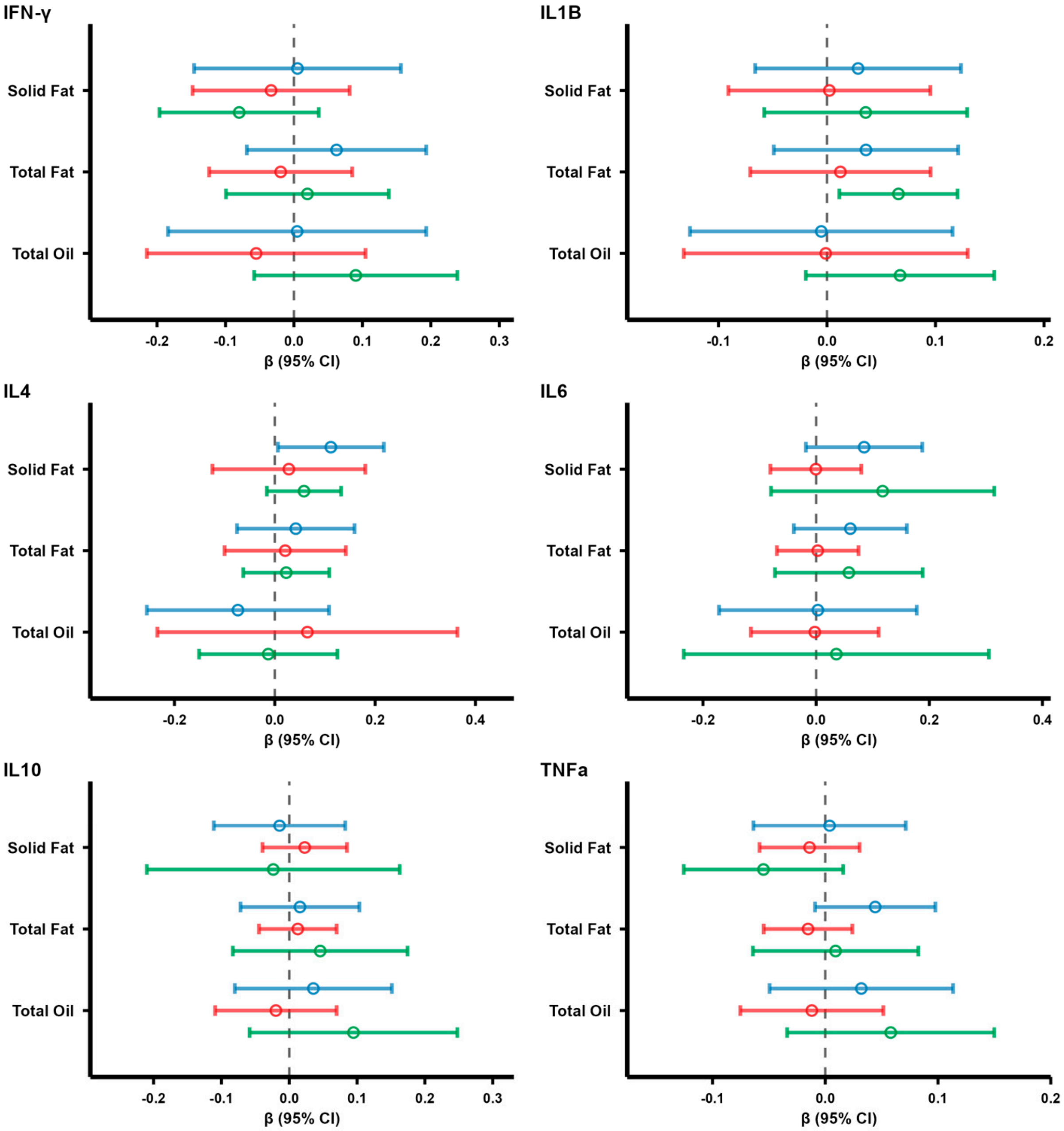

IFN-γ
Solid Fat
Total Fat
Total Oil
-0.2
-0.1
0.0
0.1
0.2
0.3
β (95% CI)
IL1B
Solid Fat
Total Fat
Total Oil
-0.1
0.0
0.1
0.2
β (95% CI)
IL4
Solid Fat
Total Fat
Total Oil
-0.2
0.0
0.2
0.4
β (95% CI)
IL6
Solid Fat
Total Fat
Total Oil
-0.2
0.0
0.2
0.4
β (95% CI)
IL10
Solid Fat
Total Fat
Total Oil
-0.2
-0.1
0.0
0.1
0.2
0.3
β (95% CI)
TNFa
Solid Fat
Total Fat
Total Oil
-0.1
0.0
0.1
0.2
β (95% CI)

Figure S1. Multivariable-adjusted linear regression models between *habitual overall fat intake* and circulating inflammatory markers at three race timepoints.

Note: Each panel displays beta coefficients (β) with 95% confidence intervals from separate multivariable-adjusted linear regression models. Inflammatory biomarkers: IFY = interferon-γ; IL-1β = interleukin-1β; IL-4 = interleukin-4; IL-6 = interleukin-6; IL-10 = interleukin-10; TNF-α = tumor necrosis factor-α. Timepoint colors: blue, Pre-Race; red, immediate post-race; green, 48-hours post-race. Filled circles and bold error bars indicate associations reaching statistical significance after Benjamini-Hochberg false discovery rate correction ($q < 0.05$). All models were adjusted for sedentary behavior, age, sex, BMI, alcohol intake, family history of high cholesterol, NSAID use, cholesterol medication use, smoking status, and blood pressure treatment.

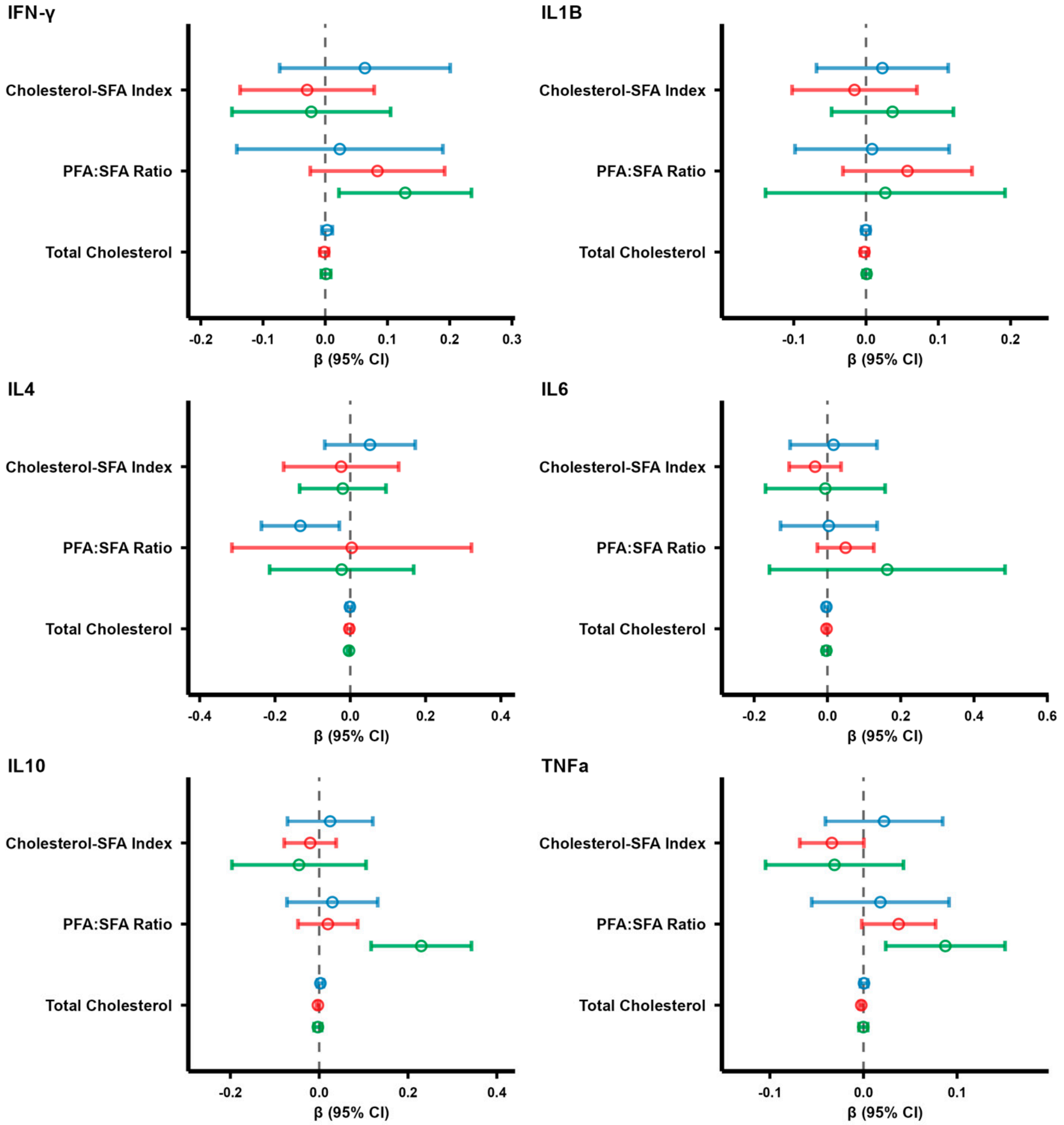
IFN-γ
Cholesterol-SFA Index
PFA:SFA Ratio
Total Cholesterol
-0.2
-0.1
0.0
0.1
0.2
0.3
β (95% CI)
IL1B
Cholesterol-SFA Index
PFA:SFA Ratio
Total Cholesterol
-0.1
0.0
0.1
0.2
β (95% CI)
IL4
Cholesterol-SFA Index
PFA:SFA Ratio
Total Cholesterol
-0.4
-0.2
0.0
0.2
0.4
β (95% CI)
IL6
Cholesterol-SFA Index
PFA:SFA Ratio
Total Cholesterol
-0.2
0.0
0.2
0.4
0.6
β (95% CI)
IL10
Cholesterol-SFA Index
PFA:SFA Ratio
Total Cholesterol
-0.2
0.0
0.2
0.4
β (95% CI)
TNFa
Cholesterol-SFA Index
PFA:SFA Ratio
Total Cholesterol
-0.1
0.0
0.1
β (95% CI)

Figure S2. Multivariable-adjusted linear regression models between habitual cholesterol-related intake and circulating inflammatory markers at three race timepoints.

Note: Each panel displays beta coefficients (β) with 95% confidence intervals from separate multivariable-adjusted linear regression models. Inflammatory biomarkers: IFY = interferon-γ; IL-1β = interleukin-1β; IL-4 = interleukin-4; IL-6 = interleukin-6; IL-10 = interleukin-10; TNF-α = tumor necrosis factor-α. Timepoint colors: blue, Pre-Race; red, immediate post-race; green, 48-hours post-race. Filled circles and bold error bars indicate associations reaching statistical significance after Benjamini-Hochberg false discovery rate correction ($q < 0.05$). All models were adjusted for sedentary behavior, age, sex, BMI, alcohol intake, family history of high cholesterol, NSAID use, cholesterol medication use, smoking status, and blood pressure treatment.

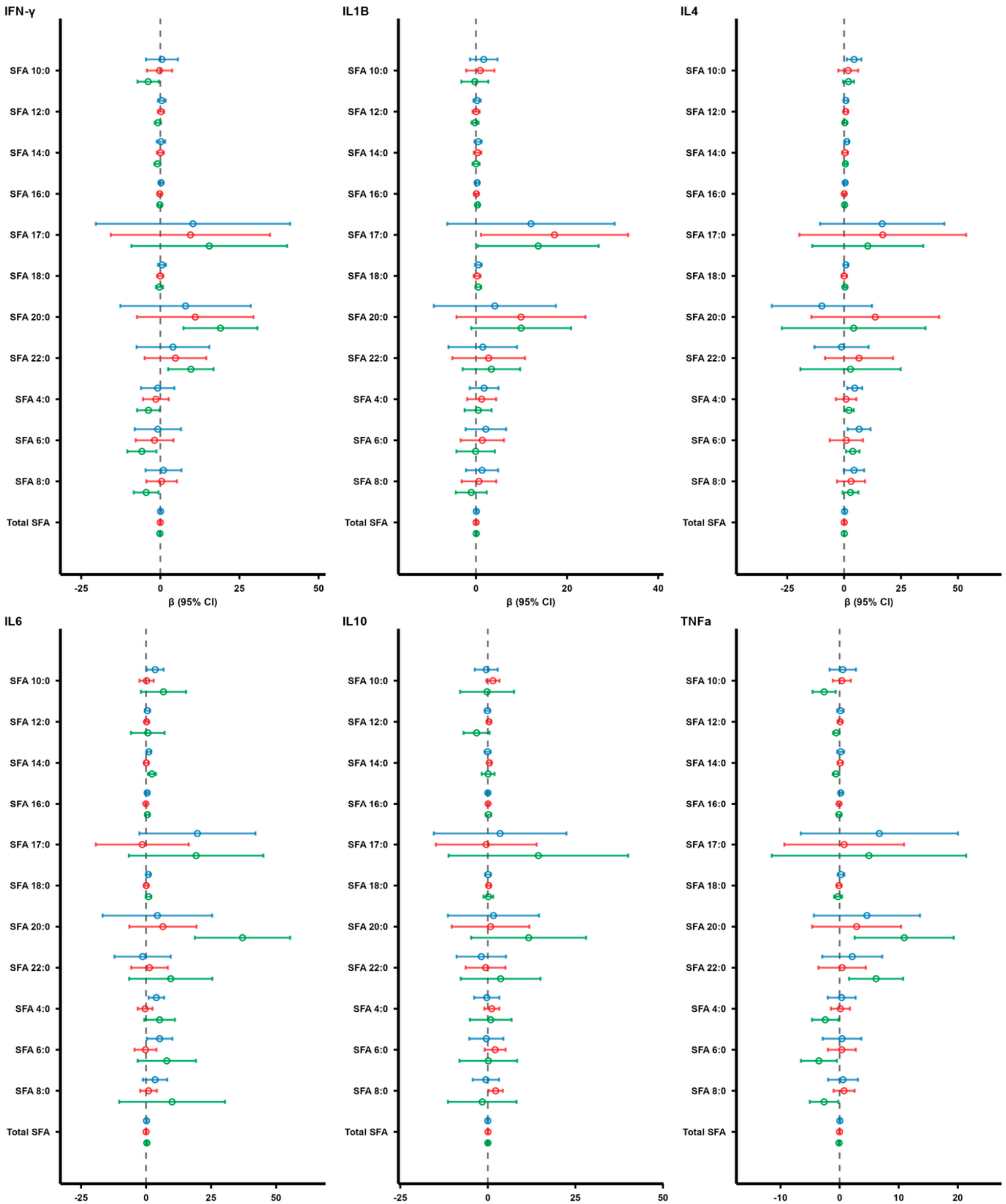
IFN-γ
IL1B
IL4
IL6
IL10
TNFa
SFA 10:0
SFA 12:0
SFA 14:0
SFA 16:0
SFA 17:0
SFA 18:0
SFA 20:0
SFA 22:0
SFA 4:0
SFA 6:0
SFA 8:0
Total SFA
β (95% CI)

Figure S3. Multivariable-adjusted linear regression models between habitual SFA-related intake and circulating inflammatory markers at three race timepoints.

Note: Each panel displays beta coefficients (β) with 95% confidence intervals from separate multivariable-adjusted linear regression models. Inflammatory biomarkers: IFY = interferon-γ; IL-1β = interleukin-1β; IL-4 = interleukin-4; IL-6 = interleukin-6; IL-10 = interleukin-10; TNF-α = tumor necrosis factor-α. Timepoint colors: blue, Pre-Race; red, immediate post-race; green, 48-hours post-race. Filled circles and bold error bars indicate associations reaching statistical significance after Benjamini-Hochberg false discovery rate correction ($q < 0.05$). All models were adjusted for sedentary behavior, age, sex, BMI, alcohol intake, family history of high cholesterol, NSAID use, cholesterol medication use, smoking status, and blood pressure treatment.

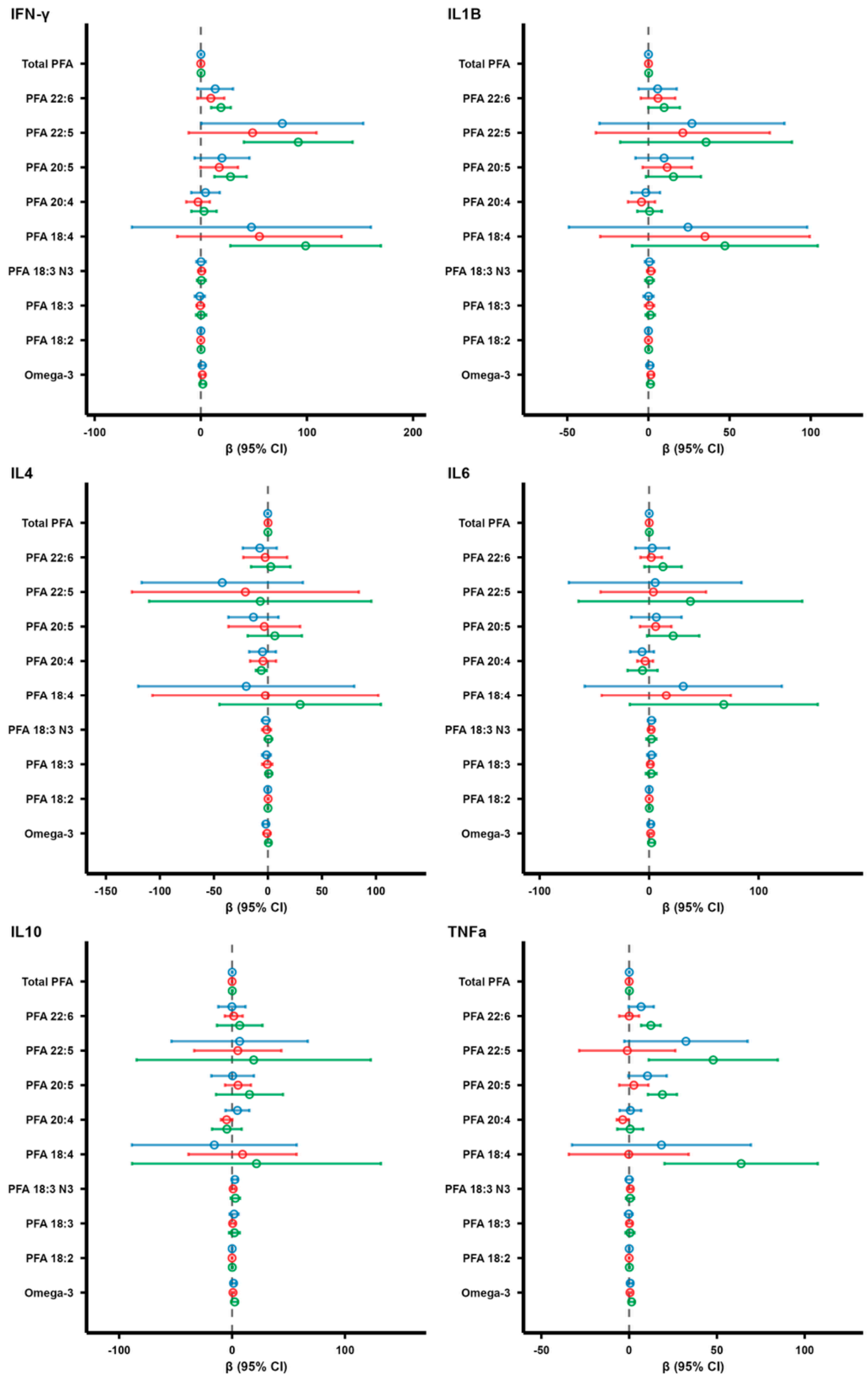

IFN-γ
IL1B
IL4
IL6
IL10
TNFa
Total PFA
PFA 22:6
PFA 22:5
PFA 20:5
PFA 20:4
PFA 18:4
PFA 18:3 N3
PFA 18:3
PFA 18:2
Omega-3
β (95% CI)

Figure S4. Multivariable-adjusted linear regression models between habitual PFA-related intake and circulating inflammatory markers at three race timepoints.
Note: Each panel displays beta coefficients (β) with 95% confidence intervals from separate multivariable-adjusted linear regression models. Inflammatory biomarkers: IFY = interferon-γ; IL-1β = interleukin-1β; IL-4 = interleukin-4; IL-6 = interleukin-6; IL-10 = interleukin-10; TNF-α = tumor necrosis factor-α. Timepoint colors: blue, Pre-Race; red, immediate post-race; green, 48-hours post-race. Filled circles and bold error bars indicate associations reaching statistical significance after Benjamini-Hochberg false discovery rate correction ($q < 0.05$). All models were adjusted for sedentary behavior, age, sex, BMI, alcohol intake, family history of high cholesterol, NSAID use, cholesterol medication use, smoking status, and blood pressure treatment.

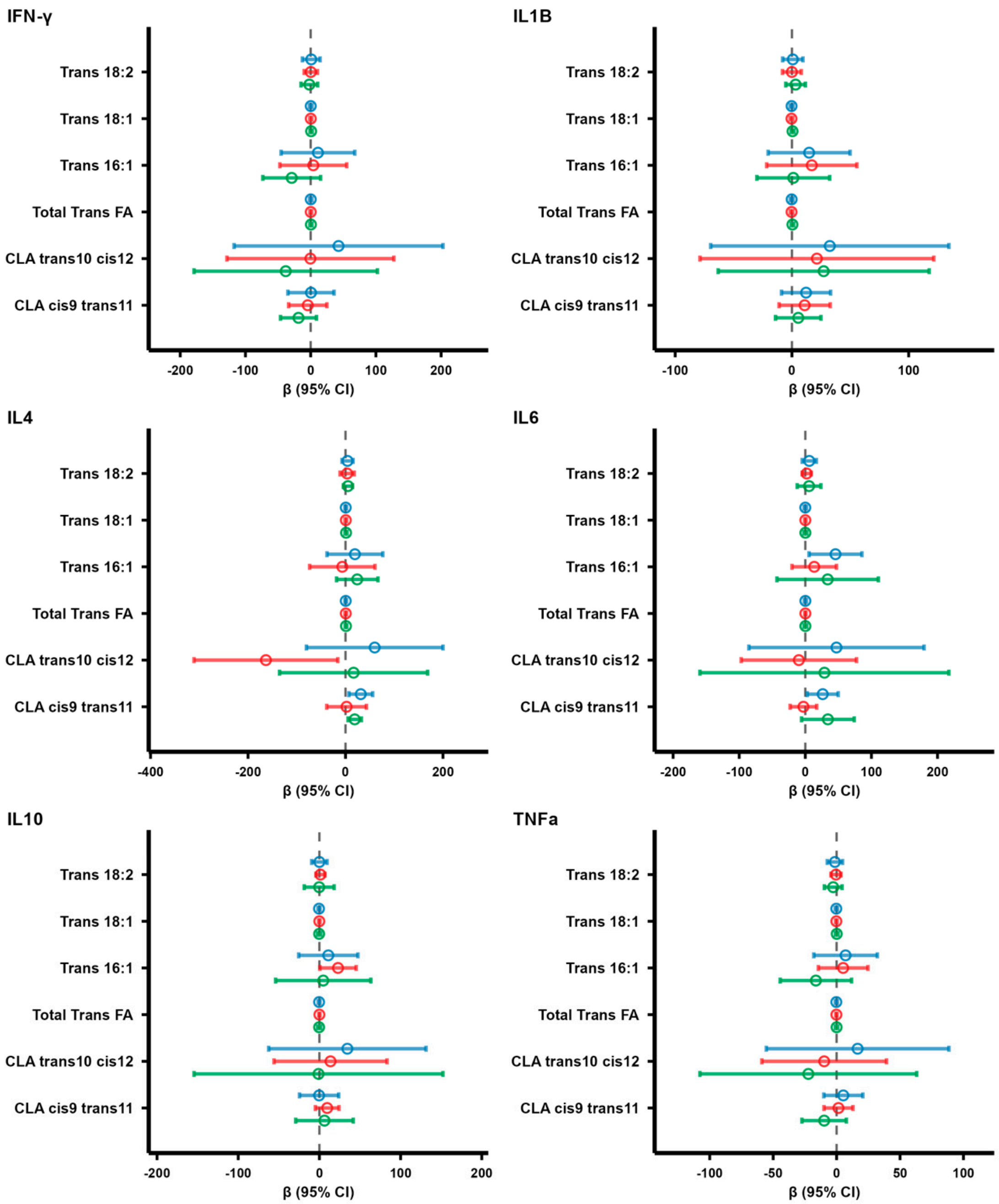

IFN-γ
IL1B
IL4
IL6
IL10
TNFa
Trans 18:2
Trans 18:1
Trans 16:1
Total Trans FA
CLA trans10 cis12
CLA cis9 trans11
β (95% CI)

Figure S5. Multivariable-adjusted linear regression models between habitual TRANs fat intake and circulating inflammatory markers at three race timepoints.

Note: Each panel displays beta coefficients (β) with 95% confidence intervals from separate multivariable-adjusted linear regression models. Inflammatory biomarkers: IFY = interferon-γ; IL-1β = interleukin-1β; IL-4 = interleukin-4; IL-6 = interleukin-6; IL-10 = interleukin-10; TNF-α = tumor necrosis factor-α. Timepoint colors: blue, Pre-Race; red, immediate post-race; green, 48-hours post-race. Filled circles and bold error bars indicate associations reaching statistical significance after Benjamini-Hochberg false discovery rate correction ($q < 0.05$). All models were adjusted for sedentary behavior, age, sex, BMI, alcohol intake, family history of high cholesterol, NSAID use, cholesterol medication use, smoking status, and blood pressure treatment.

Table S4. Multivariable-adjusted linear regression associations between habitual dietary fat intake and circulating IFN-γ at three race-related timepoints.

	Pre-Race				Immediate Post-Race				48-h Post-Race			
**Dietary Fat**	**n**	**β (95% CI)**	**p**	**q**	**n**	**β (95% CI)**	**p**	**q**	**n**	**β (95% CI)**	**p**	**q**
**Total Fats & Oil**												
Total Fat	20	0.06 (-0.07, 0.19)	0.32	0.87	19	-0.02 (-0.12, 0.09)	0.68	0.96	20	0.02 (-0.10, 0.14)	0.72	0.98
Solid Fat	20	0.01 (-0.15, 0.16)	0.94	1.00	19	-0.03 (-0.15, 0.08)	0.53	0.92	20	-0.08 (-0.20, 0.04)	0.15	0.74
Total Oil	20	0.00 (-0.18, 0.19)	0.96	1.00	19	-0.06 (-0.21, 0.10)	0.45	0.90	20	0.09 (-0.06, 0.24)	0.20	0.78
**Cholesterol**												
Total Cholesterol	20	0.00 (-0.01, 0.01)	0.44	0.90	19	-0.00 (-0.01, 0.01)	0.60	0.95	20	0.00 (-0.01, 0.01)	0.71	0.97
Cholesterol-SFA Index	20	0.06 (-0.07, 0.20)	0.32	0.88	19	-0.03 (-0.14, 0.08)	0.56	0.93	20	-0.02 (-0.15, 0.11)	0.70	0.97
PFA:SFA Ratio	20	0.02 (-0.14, 0.19)	0.76	0.98	19	0.08 (-0.02, 0.19)	0.11	0.63	20	**0.13 (0.02, 0.23)**	**0.02**	0.48
**SFA**												
Total SFA	20	0.07 (-0.19, 0.33)	0.55	0.93	19	-0.03 (-0.23, 0.17)	0.73	0.98	20	-0.14 (-0.34, 0.07)	0.16	0.76
SFA 4:0	20	-0.83 (-6.11, 4.45)	0.73	0.98	19	-1.41 (-5.47, 2.65)	0.45	0.90	20	**-3.77 (-7.36, -0.19)**	**0.04**	0.49
SFA 6:0	20	-0.79 (-8.12, 6.55)	0.82	0.99	19	-1.82 (-7.82, 4.17)	0.51	0.92	20	**-5.84 (-10.44, -1.23)**	**0.02**	0.43
SFA 8:0	20	1.00 (-4.68, 6.68)	0.70	0.97	19	0.40 (-4.43, 5.23)	0.85	0.99	20	**-4.49 (-8.42, -0.56)**	**0.03**	0.49
SFA 10:0	20	0.51 (-4.55, 5.56)	0.83	0.99	19	-0.26 (-4.28, 3.76)	0.89	0.99	20	**-3.84 (-7.22, -0.47)**	**0.03**	0.49
SFA 12:0	20	0.45 (-0.79, 1.69)	0.44	0.90	19	0.20 (-0.78, 1.17)	0.66	0.96	20	-0.77 (-1.77, 0.23)	0.12	0.64
SFA 14:0	20	0.21 (-1.13, 1.55)	0.74	0.98	19	0.02 (-1.08, 1.13)	0.96	1.00	20	-0.91 (-1.86, 0.05)	0.06	0.53
SFA 16:0	20	0.21 (-0.50, 0.92)	0.52	0.92	19	-0.19 (-0.71, 0.33)	0.43	0.90	20	-0.17 (-0.79, 0.44)	0.54	0.93
SFA 17:0	20	10.31 (-20.40, 41.02)	0.47	0.91	19	9.51 (-15.66, 34.68)	0.41	0.90	20	15.46 (-9.15, 40.06)	0.19	0.78
SFA 18:0	20	0.53 (-0.73, 1.80)	0.37	0.90	19	-0.04 (-1.03, 0.96)	0.94	1.00	20	-0.28 (-1.40, 0.84)	0.58	0.94
SFA 20:0	20	7.98 (-12.64, 28.60)	0.41	0.90	19	11.02 (-7.38, 29.42)	0.21	0.79	20	**18.99 (7.31, 30.67)**	**<0.01**	0.38
SFA 22:0	20	3.98 (-7.53, 15.49)	0.46	0.90	19	4.77 (-4.98, 14.52)	0.30	0.87	20	**9.65 (2.48, 16.81)**	**0.01**	0.43
**MFA**												
Total MFA	20	0.09 (-0.17, 0.35)	0.45	0.90	19	-0.06 (-0.27, 0.14)	0.51	0.92	20	0.11 (-0.11, 0.33)	0.30	0.87
MFA 14:1	20	11.79 (-9.36, 32.95)	0.24	0.81	19	12.08 (-2.87, 27.02)	0.10	0.62	20	9.02 (-9.37, 27.40)	0.30	0.87
MFA 16:1	20	0.28 (-3.39, 3.95)	0.87	0.99	19	-1.62 (-4.12, 0.88)	0.18	0.78	20	0.08 (-3.16, 3.32)	0.96	1.00
MFA 18:1	20	0.09 (-0.19, 0.37)	0.50	0.92	19	-0.08 (-0.30, 0.14)	0.44	0.90	20	0.11 (-0.14, 0.35)	0.35	0.90
MFA 20:1	20	7.41 (-4.62, 19.44)	0.20	0.78	19	5.96 (-3.29, 15.21)	0.18	0.78	20	**13.66 (8.94, 18.38)**	**<0.01**	**0.04**

	Pre-Race				Immediate Post-Race				48-h Post-Race			
**Dietary Fat**	**n**	**β (95% CI)**	**p**	**q**	**n**	**β (95% CI)**	**p**	**q**	**n**	**β (95% CI)**	**p**	**q**
MFA 22:1	20	34.63 (-32.32, 101.59)	0.28	0.85	19	27.39 (-21.78, 76.55)	0.24	0.81	20	**54.81 (9.27, 100.35)**	**0.02**	0.48
**PFA**												
Total PFA	20	0.09 (-0.37, 0.56)	0.66	0.96	19	0.02 (-0.38, 0.41)	0.93	1.00	20	0.26 (-0.12, 0.63)	0.15	0.74
PFA 18:2	20	0.08 (-0.43, 0.59)	0.72	0.98	19	-0.00 (-0.44, 0.44)	1.00	1.00	20	0.26 (-0.16, 0.68)	0.20	0.78
PFA 18:3	20	-0.97 (-5.73, 3.79)	0.66	0.96	19	-0.41 (-4.02, 3.20)	0.80	0.98	20	0.31 (-4.45, 5.08)	0.89	0.99
PFA 18:3 N3	20	0.21 (-4.18, 4.60)	0.92	1.00	19	0.84 (-2.43, 4.11)	0.58	0.94	20	0.53 (-3.54, 4.59)	0.78	0.98
PFA 18:4	20	47.72 (-64.92, 160.36)	0.37	0.90	19	55.25 (-22.21, 132.70)	0.14	0.71	20	**98.76 (27.83, 169.70)**	0.01	0.43
PFA 20:4	20	4.42 (-9.05, 17.88)	0.48	0.92	19	-2.57 (-13.68, 8.53)	0.61	0.96	20	2.99 (-8.95, 14.93)	0.58	0.94
PFA 20:5	20	19.98 (-5.89, 45.85)	0.12	0.63	19	**17.38 (-0.33, 35.09)**	**0.05**	**0.51**	20	**27.98 (12.83, 43.14)**	<0.01	0.26
PFA 22:5	20	**76.82 (0.47, 153.17)**	**0.05**	**0.50**	19	48.79 (-11.44, 109.02)	0.10	0.62	20	**91.85 (40.58, 143.12)**	<0.01	0.27
PFA 22:6	20	13.59 (-3.24, 30.42)	0.10	0.62	19	9.48 (-3.19, 22.15)	0.12	0.66	20	**19.03 (9.75, 28.31)**	<0.01	0.18
**Omega-3**												
Omega-3	20	1.31 (-2.06, 4.67)	0.41	0.90	19	1.47 (-0.92, 3.86)	0.20	0.78	20	1.89 (-0.81, 4.60)	0.15	0.73
**Trans FA**												
Total Trans FA	20	0.11 (-1.67, 1.89)	0.89	0.99	19	0.12 (-1.22, 1.45)	0.85	0.99	20	0.38 (-1.26, 2.01)	0.62	0.96
Trans 16:1	20	11.15 (-45.34, 67.64)	0.67	0.96	19	4.00 (-47.26, 55.27)	0.86	0.99	20	-29.07 (-73.42, 15.28)	0.17	0.78
Trans 18:1	20	0.11 (-1.87, 2.10)	0.90	0.99	19	0.17 (-1.34, 1.68)	0.80	0.98	20	0.69 (-1.09, 2.47)	0.40	0.90
Trans 18:2	20	0.89 (-12.67, 14.45)	0.89	0.99	19	0.17 (-9.97, 10.32)	0.97	1.00	20	-2.03 (-14.83, 10.77)	0.73	0.98
CLA cis9 trans11	20	0.45 (-34.90, 35.80)	0.98	1.00	19	-4.45 (-33.76, 24.86)	0.74	0.98	20	-18.67 (-46.28, 8.93)	0.16	0.76
CLA trans10 cis12	20	42.71 (-117.43, 202.84)	0.57	0.93	19	-0.41 (-128.36, 127.53)	0.99	1.00	20	-38.23 (-178.84, 102.38)	0.55	0.93
**CLA**												
CLA 18 2	20	3.26 (-27.82, 34.34)	0.82	0.99	19	-2.26 (-27.90, 23.38)	0.85	0.99	20	-12.65 (-38.26, 12.96)	0.29	0.86

β = unstandardized regression coefficient; 95% CI = 95% confidence interval; p = unadjusted p-value; q = Benjamini–Hochberg false discovery rate (FDR)-adjusted p-value; n = sample size. Bold values indicate q < 0.05. All models were adjusted for sedentary behaviour, age, sex, BMI, alcohol intake, family history of high cholesterol, NSAID use, cholesterol medication use, smoking status, and blood pressure treatment. Imm = Immediate; h = hours.

Table S5. Multivariable-adjusted linear regression associations between habitual dietary fat intake and circulating Interleukin-1β (IL-1β) at three race-related timepoints.

	Pre-Race				Immediate Post-Race				48-h Post-Race			
**Dietary Fat**	**n**	**β (95% CI)**	**p**	**q**	**n**	**β (95% CI)**	**p**	**q**	**n**	**β (95% CI)**	**p**	**q**
**Total Fats & Oil**												
Total Fat	21	0.04 (-0.05, 0.12)	0.37	0.90	20	0.01 (-0.07, 0.10)	0.74	0.98	19	**0.07 (0.01, 0.12)**	0.02	0.48
Solid Fat	21	0.03 (-0.07, 0.12)	0.52	0.92	20	0.00 (-0.09, 0.10)	0.96	1.00	19	0.04 (-0.06, 0.13)	0.41	0.90
Total Oil	21	-0.01 (-0.13, 0.12)	0.92	1.00	20	-0.00 (-0.13, 0.13)	0.98	1.00	19	0.07 (-0.02, 0.15)	0.11	0.63
**Cholesterol**												
Total Cholesterol	21	-0.00 (-0.01, 0.01)	0.91	1.00	20	-0.00 (-0.01, 0.00)	0.38	0.90	19	0.00 (-0.00, 0.01)	0.71	0.97
Cholesterol-SFA Index	21	0.02 (-0.07, 0.11)	0.59	0.94	20	-0.02 (-0.10, 0.07)	0.69	0.96	19	0.04 (-0.05, 0.12)	0.35	0.90
PFA:SFA Ratio	21	0.01 (-0.10, 0.12)	0.86	0.99	20	0.06 (-0.03, 0.15)	0.18	0.78	19	0.03 (-0.14, 0.19)	0.72	0.98
**SFA**												
Total SFA	21	0.09 (-0.06, 0.25)	0.20	0.78	20	0.04 (-0.12, 0.20)	0.59	0.94	19	0.07 (-0.08, 0.22)	0.32	0.88
SFA 4:0	21	1.80 (-1.37, 4.96)	0.23	0.81	20	1.25 (-1.95, 4.44)	0.40	0.90	19	0.49 (-2.47, 3.45)	0.71	0.97
SFA 6:0	21	2.18 (-2.28, 6.65)	0.30	0.87	20	1.40 (-3.36, 6.16)	0.52	0.92	19	-0.07 (-4.30, 4.16)	0.97	1.00
SFA 8:0	21	1.34 (-2.21, 4.89)	0.42	0.90	20	0.67 (-3.13, 4.47)	0.70	0.97	19	-1.02 (-4.43, 2.38)	0.51	0.92
SFA 10:0	21	1.71 (-1.32, 4.73)	0.24	0.81	20	0.95 (-2.16, 4.06)	0.51	0.92	19	-0.24 (-3.21, 2.73)	0.86	0.99
SFA 12:0	21	0.24 (-0.56, 1.04)	0.52	0.92	20	0.04 (-0.74, 0.83)	0.90	0.99	19	-0.21 (-0.99, 0.58)	0.56	0.93
SFA 14:0	21	0.49 (-0.30, 1.28)	0.20	0.78	20	0.36 (-0.47, 1.19)	0.35	0.90	19	0.02 (-0.75, 0.80)	0.94	1.00
SFA 16:0	21	0.27 (-0.16, 0.69)	0.19	0.78	20	0.09 (-0.34, 0.51)	0.65	0.96	19	**0.35 (0.04, 0.67)**	**0.03**	0.49

	Pre-Race				Immediate Post-Race				48-h Post-Race			
**Dietary Fat**	**n**	**β (95% CI)**	**p**	**q**	**n**	**β (95% CI)**	**p**	**q**	**n**	**β (95% CI)**	**p**	**q**
SFA 17:0	21	12.09 (-6.29, 30.46)	0.17	0.78	20	**17.25 (1.08, 33.41)**	**0.04**	0.49	19	**13.66 (0.39, 26.94)**	**0.04**	0.49
SFA 18:0	21	0.54 (-0.22, 1.30)	0.14	0.71	20	0.27 (-0.50, 1.03)	0.45	0.90	19	0.51 (-0.13, 1.15)	0.10	0.62
SFA 20:0	21	4.15 (-9.25, 17.55)	0.51	0.92	20	9.88 (-4.27, 24.04)	0.15	0.73	19	9.92 (-1.02, 20.87)	0.07	0.56
SFA 22:0	21	1.49 (-6.04, 9.02)	0.67	0.96	20	2.78 (-5.19, 10.75)	0.45	0.90	19	3.40 (-2.92, 9.71)	0.25	0.81
**MFA**												
Total MFA	21	0.03 (-0.14, 0.20)	0.75	0.98	20	-0.01 (-0.18, 0.16)	0.88	0.99	19	0.12 (0.01, 0.23)	0.03	0.49
MFA 14:1	21	9.97 (-2.82, 22.75)	0.11	0.63	20	**12.76 (2.76, 22.75)**	**0.02**	0.43	19	7.03 (-4.09, 18.16)	0.18	0.78
MFA 16:1	21	0.08 (-2.28, 2.44)	0.94	1.00	20	-0.60 (-2.76, 1.56)	0.55	0.93	19	1.29 (-0.56, 3.14)	0.15	0.73
MFA 18:1	21	0.02 (-0.17, 0.20)	0.82	0.99	20	-0.02 (-0.21, 0.16)	0.77	0.98	19	**0.13 (0.00, 0.25)**	**0.04**	0.49
MFA 20:1	21	2.99 (-5.16, 11.13)	0.43	0.90	20	4.09 (-3.46, 11.63)	0.25	0.81	19	**7.57 (1.46, 13.68)**	**0.02**	0.47
MFA 22:1	21	21.96 (-21.07, 65.00)	0.28	0.85	20	20.86 (-18.37, 60.09)	0.26	0.83	19	29.37 (-7.66, 66.40)	0.10	0.63
**PFA**												
Total PFA	21	0.01 (-0.29, 0.31)	0.95	1.00	20	0.04 (-0.27, 0.36)	0.77	0.98	19	0.16 (-0.08, 0.39)	0.17	0.77
PFA 18:2	21	0.00 (-0.33, 0.33)	0.98	1.00	20	0.03 (-0.32, 0.38)	0.84	0.99	19	0.16 (-0.10, 0.42)	0.19	0.78
PFA 18:3	21	0.08 (-3.01, 3.16)	0.96	1.00	20	0.69 (-2.13, 3.51)	0.59	0.94	19	1.13 (-1.73, 3.98)	0.39	0.90
PFA 18:3 N3	21	0.60 (-2.19, 3.38)	0.64	0.96	20	1.52 (-0.86, 3.90)	0.18	0.78	19	0.70 (-1.97, 3.36)	0.56	0.93
PFA 18:4	21	24.38 (-49.07, 97.84)	0.48	0.91	20	34.86 (-29.71, 99.43)	0.25	0.81	19	47.13 (-10.09, 104.36)	0.09	0.62
PFA 20:4	21	-1.61 (-10.41, 7.18)	0.69	0.96	20	-4.27 (-12.60, 4.06)	0.28	0.85	19	0.69 (-6.88, 8.25)	0.84	0.99

		Pre-Race				Immediate Post-Race				48-h Post-Race		
**Dietary Fat**	**n**	**β (95% CI)**	**p**	**q**	**n**	**β (95% CI)**	**p**	**q**	**n**	**β (95% CI)**	**p**	**q**
PFA 20:5	21	9.65 (-7.98, 27.29)	0.25	0.81	20	11.54 (-3.59, 26.68)	0.12	0.64	19	15.42 (-1.56, 32.40)	0.07	0.56
PFA 22:5	21	26.82 (-30.19, 83.83)	0.32	0.88	20	21.20 (-32.41, 74.81)	0.39	0.90	19	35.51 (-17.43, 88.45)	0.16	0.76
PFA 22:6	21	5.68 (-6.09, 17.45)	0.31	0.87	20	5.89 (-4.75, 16.53)	0.24	0.81	19	9.68 (-0.03, 19.39)	0.05	0.50
**Omega-3**												
Omega-3	21	0.86 (-1.30, 3.01)	0.40	0.90	20	1.50 (-0.25, 3.25)	0.08	0.61	19	1.23 (-0.80, 3.25)	0.20	0.78
**Trans FA**												
Total Trans FA	21	-0.24 (-1.37, 0.90)	0.65	0.96	20	-0.33 (-1.36, 0.70)	0.49	0.92	19	0.43 (-0.68, 1.53)	0.40	0.90
Trans 16:1	21	14.72 (-20.38, 49.82)	0.37	0.90	20	17.00 (-21.64, 55.63)	0.35	0.90	19	1.18 (-30.01, 32.36)	0.93	1.00
Trans 18:1	21	-0.31 (-1.57, 0.94)	0.59	0.94	20	-0.39 (-1.56, 0.77)	0.46	0.90	19	0.50 (-0.69, 1.70)	0.36	0.90
Trans 18:2	21	0.75 (-7.95, 9.44)	0.85	0.99	20	-0.05 (-8.09, 7.99)	0.99	1.00	19	3.12 (-5.28, 11.51)	0.42	0.90
CLA cis9 trans11	21	12.11 (-8.91, 33.13)	0.23	0.81	20	10.89 (-11.01, 32.79)	0.29	0.86	19	5.42 (-14.03, 24.87)	0.54	0.93
CLA trans10 cis12	21	32.43 (-69.60, 134.47)	0.49	0.92	20	21.36 (-78.77, 121.49)	0.64	0.96	19	27.19 (-63.21, 117.58)	0.51	0.92
**CLA**												
CLA 18 2	21	11.12 (-7.28, 29.52)	0.21	0.79	20	9.92 (-9.02, 28.87)	0.27	0.83	19	6.25 (-10.41, 22.90)	0.41	0.90

β = unstandardized regression coefficient; 95% CI = 95% confidence interval; p = unadjusted p-value; q = Benjamini–Hochberg false discovery rate (FDR)-adjusted p-value; n = sample size. Bold values indicate q < 0.05. All models were adjusted for sedentary behaviour, age, sex, BMI, alcohol intake, family history of high cholesterol, NSAID use, cholesterol medication use, smoking status, and blood pressure treatment. Imm = Immediate; h = hours.

Table S6. Multivariable-adjusted linear regression associations between habitual dietary fat intake and circulating Interleukin-4 (IL-4) at three race-related timepoints.

	Pre-Race				Immediate Post-Race				48-h Post-Race			
**Dietary Fat**	**n**	**β (95% CI)**	**p**	**q**	**n**	**β (95% CI)**	**p**	**q**	**n**	**β (95% CI)**	**p**	**q**
**Total Fats & Oil**												
Total Fat	20	0.04 (-0.08, 0.16)	0.44	0.90	15	0.02 (-0.10, 0.14)	0.66	0.96	14	0.02 (-0.06, 0.11)	0.50	0.92
Solid Fat	20	0.11 (0.01, 0.22)	0.04	0.49	15	0.03 (-0.12, 0.18)	0.64	0.96	14	0.06 (-0.02, 0.13)	0.09	0.62
Total Oil	20	-0.07 (-0.26, 0.11)	0.38	0.90	15	0.06 (-0.23, 0.36)	0.58	0.94	14	-0.01 (-0.15, 0.12)	0.81	0.98
**Cholesterol**												
Total Cholesterol	20	-0.00 (-0.01, 0.01)	0.77	0.98	15	-0.00 (-0.01, 0.01)	0.41	0.90	14	-0.00 (-0.01, 0.00)	0.09	0.62
Cholesterol-SFA Index	20	0.05 (-0.07, 0.17)	0.35	0.90	15	-0.02 (-0.18, 0.13)	0.68	0.96	14	-0.02 (-0.13, 0.09)	0.65	0.96
PFA:SFA Ratio	20	**-0.13 (-0.24, -0.03)**	**0.02**	0.43	15	0.00 (-0.31, 0.32)	0.98	1.00	14	-0.02 (-0.21, 0.17)	0.76	0.98
**SFA**												
Total SFA	20	**0.22 (0.06, 0.38)**	**0.01**	0.43	15	0.07 (-0.17, 0.31)	0.48	0.92	14	0.09 (-0.03, 0.21)	0.10	0.62
SFA 4:0	20	**4.68 (1.39, 7.98)**	**0.01**	0.43	15	0.87 (-3.65, 5.38)	0.62	0.96	14	**2.19 (0.13, 4.24)**	**0.04**	0.49
SFA 6:0	20	**6.60 (1.57, 11.63)**	**0.02**	0.43	15	0.99 (-6.33, 8.31)	0.73	0.98	14	**3.80 (0.81, 6.78)**	**0.02**	0.48
SFA 8:0	20	4.36 (-0.13, 8.85)	0.06	0.52	15	3.05 (-3.11, 9.20)	0.24	0.81	14	2.79 (-0.71, 6.30)	0.09	0.62
SFA 10:0	20	**4.39 (1.15, 7.63)**	**0.01**	0.43	15	1.81 (-2.55, 6.18)	0.31	0.87	14	1.94 (-0.46, 4.35)	0.09	0.62
SFA 12:0	20	0.79 (-0.18, 1.75)	0.10	0.62	15	0.73 (-0.25, 1.72)	0.11	0.63	14	0.33 (-0.57, 1.23)	0.37	0.90
SFA 14:0	20	**1.20 (0.32, 2.09)**	**0.01**	0.43	15	0.43 (-0.86, 1.72)	0.41	0.90	14	0.57 (-0.01, 1.15)	0.05	0.50
SFA 16:0	20	0.48 (-0.03, 0.99)	0.06	0.53	15	0.06 (-0.61, 0.73)	0.82	0.99	14	0.21 (-0.16, 0.59)	0.19	0.78
SFA 17:0	20	16.75 (-10.57, 44.07)	0.20	0.78	15	16.97 (-19.68, 53.63)	0.27	0.83	14	10.39 (-14.00, 34.78)	0.30	0.87
SFA 18:0	20	0.81 (-0.16, 1.78)	0.09	0.62	15	0.05 (-1.15, 1.24)	0.92	1.00	14	0.37 (-0.25, 0.99)	0.17	0.78
SFA 20:0	20	-9.76 (-31.78, 12.27)	0.34	0.90	15	13.66 (-14.41, 41.74)	0.25	0.81	14	4.21 (-27.36, 35.78)	0.73	0.98
SFA 22:0	20	-1.14 (-13.07, 10.79)	0.83	0.99	15	6.55 (-8.37, 21.46)	0.29	0.86	14	2.82 (-19.22, 24.86)	0.74	0.98
**MFA**												
Total MFA	20	0.00 (-0.24, 0.25)	0.98	1.00	15	0.04 (-0.31, 0.39)	0.78	0.98	14	0.00 (-0.23, 0.23)	0.98	1.00

	Pre-Race				Immediate Post-Race				48-h Post-Race			
**Dietary Fat**	**n**	**β (95% CI)**	**p**	**q**	**n**	**β (95% CI)**	**p**	**q**	**n**	**β (95% CI)**	**p**	**q**
MFA 14:1	20	7.46 (-11.87, 26.79)	0.41	0.90	15	7.13 (-23.33, 37.60)	0.55	0.93	14	5.09 (-11.86, 22.05)	0.45	0.90
MFA 16:1	20	0.99 (-2.13, 4.10)	0.49	0.92	15	-2.65 (-8.04, 2.74)	0.24	0.81	14	-0.96 (-5.44, 3.52)	0.58	0.94
MFA 18:1	20	0.00 (-0.26, 0.27)	0.97	1.00	15	0.06 (-0.31, 0.42)	0.69	0.97	14	0.00 (-0.24, 0.24)	1.00	1.00
MFA 20:1	20	-6.55 (-17.30, 4.20)	0.20	0.78	15	-0.48 (-15.67, 14.70)	0.93	1.00	14	1.60 (-12.84, 16.05)	0.77	0.98
MFA 22:1	20	-19.24 (-78.83, 40.35)	0.48	0.92	15	-0.32 (-63.16, 62.52)	0.99	1.00	14	17.53 (-27.99, 63.05)	0.35	0.90
**PFA**												
Total PFA	20	-0.18 (-0.62, 0.26)	0.38	0.90	15	0.09 (-0.40, 0.58)	0.64	0.96	14	-0.01 (-0.35, 0.34)	0.96	1.00
PFA 18:2	20	-0.14 (-0.64, 0.36)	0.54	0.93	15	0.14 (-0.37, 0.65)	0.49	0.92	14	0.00 (-0.38, 0.39)	0.98	1.00
PFA 18:3	20	-1.36 (-5.40, 2.68)	0.47	0.90	15	-0.60 (-5.29, 4.08)	0.74	0.98	14	0.72 (-2.43, 3.87)	0.56	0.93
PFA 18:3 N3	20	-1.97 (-5.50, 1.56)	0.24	0.81	15	-1.22 (-5.31, 2.88)	0.46	0.90	14	0.45 (-2.96, 3.86)	0.73	0.98
PFA 18:4	20	-20.09 (-120.12, 79.93)	0.66	0.96	15	-2.37 (-106.96, 102.21)	0.95	1.00	14	29.82 (-44.94, 104.58)	0.33	0.88
PFA 20:4	20	-4.98 (-17.39, 7.43)	0.39	0.90	15	-4.52 (-16.54, 7.51)	0.36	0.90	14	**-6.15 (-11.16, -1.14)**	**0.03**	0.49
PFA 20:5	20	-13.44 (-36.66, 9.79)	0.22	0.81	15	-3.40 (-36.60, 29.80)	0.79	0.98	14	6.38 (-18.73, 31.49)	0.52	0.92
PFA 22:5	20	-42.30 (-116.95, 32.35)	0.23	0.81	15	-20.92 (-125.96, 84.13)	0.61	0.96	14	-7.09 (-109.98, 95.79)	0.86	0.99
PFA 22:6	20	-7.56 (-23.29, 8.18)	0.31	0.87	15	-2.52 (-22.85, 17.81)	0.75	0.98	14	2.62 (-15.59, 20.83)	0.71	0.97
**Omega-3**												
Omega-3	20	-1.91 (-4.57, 0.75)	0.14	0.71	15	-0.85 (-4.15, 2.45)	0.51	0.92	14	0.42 (-2.03, 2.87)	0.66	0.96
**Trans FA**												
Total Trans FA	20	0.47 (-1.04, 1.97)	0.50	0.92	15	0.56 (-1.06, 2.18)	0.39	0.90	14	0.95 (-0.81, 2.70)	0.21	0.79

	Pre-Race				Immediate Post-Race				48-h Post-Race		
**Dietary Fat**	**n**	**β (95% CI)**	**p**	**q**	**n**	**β (95% CI)**	**p**	**q**	**n**	**β (95% CI)**	**p**	**q**
Trans 16:1	20	19.27 (-37.99, 76.52)	0.47	0.90	15	-6.60 (-73.59, 60.39)	0.80	0.98	14	24.05 (-18.46, 66.56)	0.19	0.78
Trans 18:1	20	0.47 (-1.24, 2.17)	0.55	0.93	15	0.63 (-1.09, 2.35)	0.37	0.90	14	1.13 (-1.13, 3.39)	0.24	0.81
Trans 18:2	20	4.14 (-7.11, 15.38)	0.43	0.90	15	3.32 (-11.08, 17.72)	0.56	0.93	14	5.07 (-4.23, 14.36)	0.20	0.79
CLA cis9 trans11	20	**31.35 (7.01, 55.70)**	0.02	0.43	15	2.16 (-38.51, 42.83)	0.89	0.99	14	**18.88 (5.21, 32.55)**	0.02	0.43
CLA trans10 cis12	20	59.91 (-80.15, 199.98)	0.36	0.90	15	**-163.31 (-310.81, -15.81)**	0.04	0.49	14	16.57 (-135.36, 168.51)	0.78	0.98
**CLA**												
CLA 18 2	20	23.61 (0.01, 47.21)	0.05	0.50	15	-3.77 (-42.13, 34.59)	0.80	0.98	14	14.71 (-1.18, 30.59)	0.06	0.53

β = unstandardized regression coefficient; 95% CI = 95% confidence interval; p = unadjusted p-value; q = Benjamini–Hochberg false discovery rate (FDR)-adjusted p-value; n = sample size. Bold values indicate q < 0.05. All models were adjusted for sedentary behaviour, age, sex, BMI, alcohol intake, family history of high cholesterol, NSAID use, cholesterol medication use, smoking status, and blood pressure treatment. Imm = Immediate; h = hours.

Table S7. Multivariable-adjusted linear regression associations between habitual dietary fat intake and circulating Interleukin-6 (IL-6) at three race-related timepoints.

	Pre-Race				Immediate Post-Race				48-h Post-Race			
**Dietary Fat**	**n**	**β (95% CI)**	**p**	**q**	**n**	**β (95% CI)**	**p**	**q**	**n**	**β (95% CI)**	**p**	**q**
**Total Fats & Oil**												
Total Fat	18	0.06 (-0.04, 0.16)	0.20	0.78	19	0.00 (-0.07, 0.07)	0.93	1.00	16	0.06 (-0.07, 0.19)	0.31	0.87
Solid Fat	18	0.08 (-0.02, 0.19)	0.09	0.62	19	-0.00 (-0.08, 0.08)	0.99	1.00	16	0.12 (-0.08, 0.31)	0.19	0.78
Total Oil	18	0.00 (-0.17, 0.18)	0.97	1.00	19	-0.00 (-0.12, 0.11)	0.96	1.00	16	0.04 (-0.23, 0.31)	0.75	0.98
**Cholesterol**												
Total Cholesterol	18	-0.00 (-0.01, 0.00)	0.38	0.90	19	-0.00 (-0.01, 0.00)	0.24	0.81	16	-0.00 (-0.01, 0.01)	0.44	0.90
Cholesterol-SFA Index	18	0.02 (-0.10, 0.14)	0.75	0.98	19	-0.03 (-0.10, 0.04)	0.31	0.87	16	-0.01 (-0.17, 0.16)	0.93	1.00
PFA:SFA Ratio	18	0.00 (-0.13, 0.14)	0.95	1.00	19	0.05 (-0.03, 0.13)	0.18	0.78	16	0.16 (-0.16, 0.48)	0.25	0.81
**SFA**												
Total SFA	18	**0.18 (0.02, 0.33)**	**0.03**	0.49	19	0.00 (-0.14, 0.14)	0.98	1.00	16	**0.30 (0.01, 0.59)**	**0.04**	0.49
SFA 4:0	18	**3.95 (0.95, 6.95)**	**0.02**	0.43	19	-0.36 (-3.22, 2.50)	0.78	0.98	16	5.21 (-0.69, 11.12)	0.07	0.57
SFA 6:0	18	**5.23 (0.31, 10.14)**	**0.04**	0.49	19	-0.23 (-4.44, 3.98)	0.90	0.99	16	7.98 (-3.24, 19.20)	0.13	0.66
SFA 8:0	18	3.47 (-1.21, 8.15)	0.13	0.66	19	0.96 (-2.27, 4.19)	0.52	0.92	16	10.02 (-10.34, 30.38)	0.26	0.83
SFA 10:0	18	**3.50 (0.25, 6.75)**	**0.04**	0.49	19	0.20 (-2.55, 2.95)	0.87	0.99	16	6.69 (-2.00, 15.38)	0.10	0.63
SFA 12:0	18	0.51 (-0.51, 1.52)	0.28	0.85	19	0.19 (-0.46, 0.85)	0.52	0.92	16	0.66 (-5.83, 7.14)	0.80	0.98
SFA 14:0	18	**1.07 (0.27, 1.86)**	**0.01**	0.43	19	0.11 (-0.64, 0.86)	0.75	0.98	16	**2.26 (0.70, 3.81)**	**0.01**	0.43
SFA 16:0	18	0.42 (-0.03, 0.88)	0.07	0.54	19	-0.07 (-0.44, 0.29)	0.67	0.96	16	0.52 (-0.15, 1.18)	0.10	0.62
SFA 17:0	18	19.75 (-2.59, 42.08)	0.08	0.58	19	-1.47 (-19.34, 16.40)	0.86	0.99	16	19.25 (-6.62, 45.13)	0.11	0.63
SFA 18:0	18	**0.88 (0.12, 1.64)**	**0.03**	0.49	19	0.07 (-0.61, 0.75)	0.83	0.99	16	0.96 (-0.15, 2.06)	0.08	0.58
SFA 20:0	18	4.36 (-16.73, 25.44)	0.65	0.96	19	6.48 (-6.44, 19.40)	0.29	0.86	16	**37.09 (18.75, 55.42)**	<0.01	0.28

	Pre-Race				Immediate Post-Race				48-h Post-Race			
**Dietary Fat**	**n**	**β (95% CI)**	**p**	**q**	**n**	**β (95% CI)**	**p**	**q**	**n**	**β (95% CI)**	**p**	**q**
SFA 22:0	18	-1.33 (-12.18, 9.51)	0.78	0.98	19	1.32 (-5.72, 8.36)	0.68	0.96	16	9.50 (-6.51, 25.51)	0.19	0.78
**MFA**												
Total MFA	18	0.02 (-0.20, 0.25)	0.81	0.98	19	-0.03 (-0.17, 0.12)	0.67	0.96	16	0.07 (-0.25, 0.40)	0.59	0.94
MFA 14:1	18	**14.50 (0.42, 28.58)**	**0.04**	0.49	19	2.58 (-9.23, 14.40)	0.63	0.96	16	9.82 (-8.88, 28.51)	0.24	0.81
MFA 16:1	18	-0.23 (-3.21, 2.76)	0.87	0.99	19	-1.38 (-2.98, 0.22)	0.08	0.60	16	0.07 (-5.89, 6.04)	0.98	1.00
MFA 18:1	18	0.02 (-0.23, 0.26)	0.87	0.99	19	-0.03 (-0.19, 0.12)	0.65	0.96	16	0.07 (-0.29, 0.43)	0.66	0.96
MFA 20:1	18	1.22 (-9.54, 11.98)	0.80	0.98	19	1.77 (-5.13, 8.68)	0.58	0.94	16	11.35 (-0.90, 23.61)	0.06	0.53
MFA 22:1	18	19.23 (-34.54, 73.00)	0.43	0.90	19	2.55 (-33.88, 38.98)	0.88	0.99	16	49.84 (-0.38, 100.07)	0.05	0.50
**PFA**												
Total PFA	18	0.09 (-0.34, 0.53)	0.63	0.96	19	0.11 (-0.15, 0.37)	0.36	0.90	16	0.19 (-0.42, 0.80)	0.46	0.90
PFA 18:2	18	0.10 (-0.39, 0.59)	0.64	0.96	19	0.11 (-0.18, 0.40)	0.43	0.90	16	0.21 (-0.49, 0.90)	0.48	0.92
PFA 18:3	18	2.18 (-1.81, 6.17)	0.24	0.81	19	1.11 (-1.23, 3.44)	0.31	0.87	16	1.90 (-3.02, 6.82)	0.37	0.90
PFA 18:3 N3	18	2.21 (-1.11, 5.52)	0.16	0.77	19	**1.89 (0.11, 3.67)**	**0.04**	0.49	16	2.07 (-2.54, 6.69)	0.30	0.87
PFA 18:4	18	31.20 (-58.90, 121.31)	0.45	0.90	19	15.67 (-43.33, 74.66)	0.56	0.93	16	68.20 (-17.68, 154.08)	0.10	0.62
PFA 20:4	18	-6.42 (-17.28, 4.44)	0.21	0.79	19	-3.61 (-10.82, 3.60)	0.29	0.86	16	-6.01 (-19.70, 7.67)	0.31	0.87
PFA 20:5	18	6.61 (-16.42, 29.64)	0.53	0.92	19	6.00 (-8.37, 20.38)	0.37	0.90	16	21.98 (-1.90, 45.87)	0.06	0.54
PFA 22:5	18	5.53 (-73.36, 84.43)	0.88	0.99	19	3.86 (-44.34, 52.06)	0.86	0.99	16	37.73 (-64.45, 139.91)	0.39	0.90
PFA 22:6	18	2.83 (-12.61, 18.27)	0.68	0.96	19	1.94 (-7.91, 11.78)	0.67	0.96	16	12.71 (-4.30, 29.72)	0.11	0.63
**Omega-3**												
Omega-3	18	1.51 (-1.02, 4.03)	0.21	0.79	19	**1.44 (0.01, 2.88)**	0.05	0.50	16	2.26 (-0.71, 5.23)	0.11	0.63
**Trans FA**												

	Pre-Race				Immediate Post-Race				48-h Post-Race			
**Dietary Fat**	**n**	**β (95% CI)**	**p**	**q**	**n**	**β (95% CI)**	**p**	**q**	**n**	**β (95% CI)**	**p**	**q**
Total Trans FA	18	0.08 (-1.44, 1.60)	0.90	0.99	19	0.08 (-0.84, 0.99)	0.85	0.99	16	0.12 (-1.93, 2.17)	0.88	0.99
Trans 16:1	18	**45.64 (5.63, 85.65)**	**0.03**	0.49	19	13.49 (-20.14, 47.12)	0.39	0.90	16	33.83 (-42.89, 110.54)	0.31	0.87
Trans 18:1	18	-0.05 (-1.79, 1.69)	0.95	1.00	19	0.04 (-0.99, 1.08)	0.93	1.00	16	0.10 (-2.17, 2.36)	0.92	1.00
Trans 18:2	18	5.99 (-4.67, 16.66)	0.23	0.81	19	2.11 (-4.65, 8.86)	0.50	0.92	16	5.67 (-12.28, 23.62)	0.45	0.90
CLA cis9 trans11	18	**26.34 (2.92, 49.76)**	**0.03**	0.49	19	-2.87 (-22.94, 17.20)	0.75	0.98	16	34.14 (-5.74, 74.02)	0.08	0.59
CLA trans10 cis12	18	47.13 (-85.36, 179.61)	0.44	0.90	19	-9.79 (-97.01, 77.44)	0.81	0.98	16	28.94 (-159.36, 217.24)	0.71	0.97
**CLA**												
CLA 18 2	18	**21.71 (0.46, 42.96)**	0.05	0.49	19	-2.10 (-19.61, 15.41)	0.79	0.98	16	26.59 (-6.52, 59.69)	0.09	0.62

β = unstandardized regression coefficient; 95% CI = 95% confidence interval; p = unadjusted p-value; q = Benjamini–Hochberg false discovery rate (FDR)-adjusted p-value; n = sample size. Bold values indicate q < 0.05. All models were adjusted for sedentary behaviour, age, sex, BMI, alcohol intake, family history of high cholesterol, NSAID use, cholesterol medication use, smoking status, and blood pressure treatment. Imm = Immediate; h = hours.

Table S8. Multivariable-adjusted linear regression associations between habitual dietary fat intake and circulating Interleukin-10 (IL-10) at three race-related timepoints.

	Pre-Race				Immediate Post-Race				48-h Post-Race			
**Dietary Fat**	**n**	**β (95% CI)**	**p**	**q**	**n**	**β (95% CI)**	**p**	**q**	**n**	**β (95% CI)**	**p**	**q**
**Total Fats & Oil**												
Total Fat	18	0.02 (-0.07, 0.10)	0.69	0.96	19	0.01 (-0.04, 0.07)	0.63	0.96	17	0.05 (-0.08, 0.17)	0.42	0.90
Solid Fat	18	-0.01 (-0.11, 0.08)	0.74	0.98	19	0.02 (-0.04, 0.09)	0.43	0.90	17	-0.02 (-0.21, 0.16)	0.77	0.98
Total Oil	18	0.04 (-0.08, 0.15)	0.50	0.92	19	-0.02 (-0.11, 0.07)	0.63	0.96	17	0.09 (-0.06, 0.25)	0.18	0.78
**Cholesterol**												
Total Cholesterol	18	0.00 (-0.00, 0.01)	0.37	0.90	19	-0.00 (-0.01, 0.00)	0.07	0.57	17	-0.00 (-0.01, 0.01)	0.43	0.90
Cholesterol-SFA Index	18	0.02 (-0.07, 0.12)	0.57	0.93	19	-0.02 (-0.08, 0.04)	0.45	0.90	17	-0.05 (-0.20, 0.11)	0.49	0.92
PFA:SFA Ratio	18	0.03 (-0.07, 0.13)	0.52	0.92	19	0.02 (-0.05, 0.09)	0.53	0.92	17	**0.23 (0.12, 0.34)**	<0.01	0.26
**SFA**												
Total SFA	18	-0.01 (-0.18, 0.16)	0.88	0.99	19	0.06 (-0.04, 0.16)	0.22	0.81	17	0.02 (-0.34, 0.38)	0.89	0.99
SFA 4:0	18	-0.30 (-3.91, 3.31)	0.85	0.99	19	1.13 (-1.02, 3.28)	0.26	0.83	17	0.83 (-5.15, 6.80)	0.75	0.98
SFA 6:0	18	-0.43 (-5.31, 4.45)	0.84	0.99	19	2.08 (-0.92, 5.08)	0.15	0.74	17	0.13 (-8.12, 8.37)	0.97	1.00
SFA 8:0	18	-0.55 (-4.31, 3.20)	0.74	0.98	19	**2.23 (0.16, 4.29)**	**0.04**	0.49	17	-1.59 (-11.38, 8.20)	0.70	0.97
SFA 10:0	18	-0.46 (-3.72, 2.81)	0.76	0.98	19	1.45 (-0.47, 3.38)	0.12	0.65	17	-0.21 (-7.92, 7.49)	0.95	1.00
SFA 12:0	18	-0.12 (-0.93, 0.69)	0.74	0.98	19	0.32 (-0.16, 0.81)	0.16	0.77	17	-3.21 (-6.94, 0.53)	0.08	0.59
SFA 14:0	18	-0.07 (-0.95, 0.81)	0.86	0.99	19	0.45 (-0.05, 0.95)	0.07	0.57	17	0.09 (-1.76, 1.94)	0.91	1.00
SFA 16:0	18	0.02 (-0.46, 0.49)	0.94	1.00	19	0.08 (-0.21, 0.37)	0.55	0.93	17	0.17 (-0.63, 0.97)	0.62	0.96

	Pre-Race				Immediate Post-Race				48-h Post-Race			
**Dietary Fat**	**n**	**β (95% CI)**	**p**	**q**	**n**	**β (95% CI)**	**p**	**q**	**n**	**β (95% CI)**	**p**	**q**
SFA 17:0	18	3.51 (-15.43, 22.45)	0.68	0.96	19	-0.45 (-14.84, 13.94)	0.95	1.00	17	14.43 (-11.21, 40.07)	0.22	0.80
SFA 18:0	18	0.07 (-0.77, 0.90)	0.86	0.99	19	0.24 (-0.27, 0.76)	0.32	0.87	17	0.15 (-1.24, 1.54)	0.80	0.98
SFA 20:0	18	1.60 (-11.42, 14.62)	0.78	0.98	19	0.77 (-10.31, 11.86)	0.88	0.99	17	11.66 (-4.77, 28.08)	0.13	0.68
SFA 22:0	18	-1.86 (-8.93, 5.22)	0.56	0.93	19	-0.64 (-6.34, 5.05)	0.80	0.98	17	3.65 (-7.74, 15.04)	0.46	0.90
**MFA**												
Total MFA	18	0.03 (-0.14, 0.19)	0.73	0.98	19	-0.01 (-0.13, 0.11)	0.85	0.99	17	0.11 (-0.15, 0.37)	0.35	0.90
MFA 14:1	18	5.85 (-7.08, 18.78)	0.33	0.88	19	2.03 (-7.47, 11.53)	0.64	0.96	17	8.09 (-10.64, 26.82)	0.33	0.88
MFA 16:1	18	0.17 (-2.12, 2.46)	0.87	0.99	19	-0.75 (-2.17, 0.68)	0.27	0.83	17	-0.46 (-6.15, 5.23)	0.85	0.99
MFA 18:1	18	0.02 (-0.16, 0.20)	0.79	0.98	19	-0.01 (-0.14, 0.11)	0.81	0.99	17	0.11 (-0.18, 0.40)	0.39	0.90
MFA 20:1	18	0.17 (-7.87, 8.21)	0.96	1.00	19	0.56 (-5.08, 6.20)	0.83	0.99	17	7.26 (-5.46, 19.98)	0.21	0.79
MFA 22:1	18	-13.26 (-56.93, 30.41)	0.50	0.92	19	6.23 (-22.71, 35.18)	0.64	0.96	17	14.10 (-57.92, 86.13)	0.65	0.96
**PFA**												
Total PFA	18	0.08 (-0.21, 0.37)	0.54	0.93	19	-0.01 (-0.23, 0.20)	0.89	0.99	17	0.16 (-0.24, 0.56)	0.37	0.90
PFA 18:2	18	0.06 (-0.26, 0.38)	0.68	0.96	19	-0.02 (-0.26, 0.22)	0.84	0.99	17	0.16 (-0.29, 0.61)	0.41	0.90
PFA 18:3	18	1.76 (-2.16, 5.68)	0.33	0.88	19	0.53 (-1.42, 2.48)	0.55	0.93	17	2.05 (-2.72, 6.82)	0.33	0.88
PFA 18:3 N3	18	**2.50 (0.02, 4.98)**	**0.05**	0.50	19	0.96 (-0.73, 2.64)	0.23	0.81	17	2.92 (-1.16, 7.00)	0.13	0.67
PFA 18:4	18	-15.74 (-88.71, 57.24)	0.63	0.96	19	9.27 (-38.59, 57.13)	0.67	0.96	17	21.61 (-88.57, 131.79)	0.65	0.96
PFA 20:4	18	4.73 (-5.82, 15.29)	0.33	0.88	19	-4.85 (-9.86, 0.15)	0.06	0.52	17	-4.58 (-17.71, 8.55)	0.43	0.90

	Pre-Race				Immediate Post-Race				48-h Post-Race			
**Dietary Fat**	**n**	**β (95% CI)**	**p**	**q**	**n**	**β (95% CI)**	**p**	**q**	**n**	**β (95% CI)**	**p**	**q**
PFA 20:5	18	0.49 (-18.37, 19.36)	0.95	1.00	19	5.27 (-6.17, 16.72)	0.32	0.88	17	15.44 (-14.22, 45.10)	0.25	0.81
PFA 22:5	18	6.71 (-53.69, 67.11)	0.80	0.98	19	5.07 (-33.56, 43.69)	0.77	0.98	17	19.16 (-84.51, 122.84)	0.67	0.96
PFA 22:6	18	-0.14 (-12.20, 11.92)	0.98	1.00	19	1.50 (-6.42, 9.42)	0.68	0.96	17	6.77 (-13.41, 26.95)	0.44	0.90
**Omega-3**												
Omega-3	18	1.29 (-0.81, 3.40)	0.19	0.78	19	0.82 (-0.49, 2.13)	0.19	0.78	17	2.30 (-0.73, 5.33)	0.11	0.63
**Trans FA**												
Total Trans FA	18	-0.41 (-1.53, 0.71)	0.43	0.90	19	-0.04 (-0.77, 0.70)	0.92	1.00	17	-0.37 (-2.27, 1.53)	0.65	0.96
Trans 16:1	18	10.96 (-25.52, 47.44)	0.51	0.92	19	**22.90 (0.55, 45.25)**	0.05	0.49	17	4.72 (-53.92, 63.37)	0.85	0.99
Trans 18:1	18	-0.50 (-1.74, 0.74)	0.38	0.90	19	-0.12 (-0.95, 0.71)	0.75	0.98	17	-0.34 (-2.37, 1.70)	0.70	0.97
Trans 18:2	18	-0.02 (-9.44, 9.40)	1.00	1.00	19	1.27 (-4.23, 6.76)	0.62	0.96	17	-0.26 (-18.69, 18.18)	0.97	1.00
CLA cis9 trans11	18	-0.39 (-24.51, 23.72)	0.97	1.00	19	9.57 (-4.97, 24.10)	0.17	0.78	17	6.26 (-29.17, 41.68)	0.68	0.96
CLA trans10 cis12	18	34.42 (-62.51, 131.35)	0.44	0.90	19	13.68 (-55.93, 83.28)	0.67	0.96	17	-1.12 (-154.44, 152.20)	0.99	1.00
**CLA**												
CLA 18 2	18	1.51 (-19.16, 22.18)	0.87	0.99	19	7.61 (-5.31, 20.52)	0.22	0.80	17	5.18 (-24.77, 35.14)	0.69	0.96

β = unstandardized regression coefficient; 95% CI = 95% confidence interval; p = unadjusted p-value; q = Benjamini–Hochberg false discovery rate (FDR)-adjusted p-value; n = sample size. Bold values indicate q < 0.05. All models were adjusted for sedentary behaviour, age, sex, BMI, alcohol intake, family history of high cholesterol, NSAID use, cholesterol medication use, smoking status, and blood pressure treatment. Imm = Immediate; h = hours.

Table S9. Multivariable-adjusted linear regression associations between habitual dietary fat intake and circulating Tumor Necrosis Factor-α (TNF-α) at three race-related timepoints.

	Pre-Race				Immediate Post-Race				48-h Post-Race			
**Dietary Fat**	**n**	**β (95% CI)**	**p**	**q**	**n**	**β (95% CI)**	**p**	**q**	**n**	**β (95% CI)**	**p**	**q**
**Total Fats & Oil**												
Total Fat	20	0.04 (-0.01, 0.10)	0.09	0.62	19	-0.02 (-0.05, 0.02)	0.40	0.90	21	0.01 (-0.06, 0.08)	0.79	0.98
Solid Fat	20	0.00 (-0.06, 0.07)	0.90	0.99	19	-0.01 (-0.06, 0.03)	0.50	0.92	21	-0.05 (-0.13, 0.02)	0.11	0.63
Total Oil	20	0.03 (-0.05, 0.11)	0.40	0.90	19	-0.01 (-0.08, 0.05)	0.68	0.96	21	0.06 (-0.03, 0.15)	0.19	0.78
**Cholesterol**												
Total Cholesterol	20	0.00 (-0.00, 0.00)	0.76	0.98	19	**-0.00 (-0.00, -0.00)**	**0.03**	**0.49**	21	-0.00 (-0.00, 0.00)	0.92	1.00
Cholesterol-SFA Index	20	0.02 (-0.04, 0.08)	0.45	0.90	19	**-0.03 (-0.07, 0.00)**	**0.05**	**0.50**	21	-0.03 (-0.10, 0.04)	0.37	0.90
PFA:SFA Ratio	20	0.02 (-0.06, 0.09)	0.60	0.94	19	0.04 (-0.00, 0.08)	0.06	0.53	21	**0.09 (0.02, 0.15)**	**0.01**	0.43
**SFA**												
Total SFA	20	0.05 (-0.06, 0.16)	0.35	0.90	19	-0.01 (-0.09, 0.07)	0.77	0.98	21	-0.10 (-0.22, 0.03)	0.11	0.63
SFA 4:0	20	0.35 (-2.01, 2.72)	0.75	0.98	19	0.14 (-1.49, 1.76)	0.85	0.99	21	**-2.43 (-4.68, -0.17)**	**0.04**	0.49
SFA 6:0	20	0.42 (-2.86, 3.70)	0.78	0.98	19	0.37 (-2.01, 2.74)	0.73	0.98	21	**-3.50 (-6.55, -0.45)**	**0.03**	0.49
SFA 8:0	20	0.57 (-1.95, 3.10)	0.62	0.96	19	0.76 (-1.03, 2.55)	0.36	0.90	21	**-2.62 (-5.05, -0.20)**	**0.04**	0.49
SFA 10:0	20	0.54 (-1.70, 2.77)	0.61	0.95	19	0.38 (-1.15, 1.92)	0.59	0.94	21	**-2.62 (-4.59, -0.65)**	**0.01**	0.43
SFA 12:0	20	0.14 (-0.42, 0.70)	0.59	0.94	19	0.09 (-0.28, 0.47)	0.59	0.94	21	**-0.57 (-1.12, -0.01)**	**0.05**	0.49
SFA 14:0	20	0.17 (-0.42, 0.76)	0.53	0.93	19	0.11 (-0.30, 0.53)	0.55	0.93	21	**-0.61 (-1.18, -0.03)**	**0.04**	0.49
SFA 16:0	20	0.19 (-0.10, 0.49)	0.17	0.78	19	-0.09 (-0.29, 0.10)	0.31	0.87	21	-0.11 (-0.49, 0.27)	0.53	0.93

	Pre-Race				Immediate Post-Race				48-h Post-Race			
**Dietary Fat**	**n**	**β (95% CI)**	**p**	**q**	**n**	**β (95% CI)**	**p**	**q**	**n**	**β (95% CI)**	**p**	**q**
SFA 17:0	20	6.73 (-6.57, 20.03)	0.29	0.86	19	0.78 (-9.36, 10.92)	0.87	0.99	21	4.97 (-11.48, 21.43)	0.52	0.92
SFA 18:0	20	0.25 (-0.32, 0.81)	0.35	0.90	19	-0.10 (-0.48, 0.28)	0.55	0.93	21	-0.25 (-0.93, 0.43)	0.43	0.90
SFA 20:0	20	4.62 (-4.37, 13.62)	0.28	0.85	19	2.87 (-4.66, 10.40)	0.41	0.90	21	**10.94 (2.54, 19.33)**	**0.02**	0.43
SFA 22:0	20	2.14 (-2.94, 7.22)	0.37	0.90	19	0.43 (-3.59, 4.45)	0.81	0.99	21	**6.18 (1.60, 10.75)**	**0.01**	0.43
**MFA**												
Total MFA	20	0.09 (-0.02, 0.19)	0.10	0.62	19	-0.04 (-0.12, 0.04)	0.27	0.83	21	0.07 (-0.06, 0.21)	0.25	0.81
MFA 14:1	20	3.86 (-5.94, 13.66)	0.40	0.90	19	2.19 (-4.40, 8.78)	0.47	0.91	21	0.66 (-11.44, 12.77)	0.91	0.99
MFA 16:1	20	0.59 (-1.00, 2.18)	0.43	0.90	19	**-0.94 (-1.75, -0.12)**	**0.03**	**0.49**	21	0.17 (-1.79, 2.12)	0.85	0.99
MFA 18:1	20	0.09 (-0.03, 0.20)	0.11	0.63	19	-0.05 (-0.13, 0.04)	0.24	0.81	21	0.07 (-0.07, 0.22)	0.29	0.86
MFA 20:1	20	4.12 (-0.98, 9.22)	0.10	0.62	19	0.25 (-3.74, 4.24)	0.89	0.99	21	**9.14 (6.43, 11.86)**	**<0.01**	**0.02**
MFA 22:1	20	21.20 (-6.97, 49.38)	0.12	0.66	19	-1.24 (-21.92, 19.44)	0.90	0.99	21	39.12 (12.99, 65.26)	<0.01	0.43
**PFA**												
Total PFA	20	0.05 (-0.16, 0.26)	0.61	0.96	19	-0.01 (-0.17, 0.14)	0.84	0.99	21	0.13 (-0.11, 0.36)	0.25	0.81
PFA 18:2	20	0.05 (-0.18, 0.27)	0.66	0.96	19	-0.02 (-0.19, 0.15)	0.79	0.98	21	0.12 (-0.14, 0.38)	0.33	0.88
PFA 18:3	20	-0.23 (-2.37, 1.92)	0.82	0.99	19	0.18 (-1.22, 1.58)	0.78	0.98	21	0.48 (-2.06, 3.02)	0.68	0.96
PFA 18:3 N3	20	0.02 (-1.95, 1.99)	0.98	1.00	19	0.67 (-0.52, 1.86)	0.23	0.81	21	0.52 (-1.79, 2.83)	0.63	0.96
PFA 18:4	20	18.40 (-32.58, 69.38)	0.44	0.90	19	-0.25 (-34.39, 33.89)	0.99	1.00	21	**63.79 (20.12, 107.45)**	**<0.01**	0.43
PFA 20:4	20	0.70 (-5.46, 6.87)	0.80	0.98	19	**-3.67 (-7.06, -0.29)**	**0.04**	**0.49**	21	0.58 (-6.77, 7.94)	0.86	0.99

	Pre-Race				Immediate Post-Race				48-h Post-Race			
**Dietary Fat**	**n**	**β (95% CI)**	**p**	**q**	**n**	**β (95% CI)**	**p**	**q**	**n**	**β (95% CI)**	**p**	**q**
PFA 20:5	20	10.44 (-0.49, 21.38)	0.06	0.53	19	2.67 (-5.64, 10.98)	0.49	0.92	21	**18.95 (10.70, 27.21)**	**<0.01**	0.10
PFA 22:5	20	32.34 (-2.79, 67.48)	0.07	0.55	19	-1.07 (-28.46, 26.31)	0.93	1.00	21	**47.89 (11.15, 84.64)**	**0.02**	0.43
PFA 22:6	20	6.82 (-0.39, 14.03)	0.06	0.53	19	0.00 (-5.65, 5.65)	1.00	1.00	21	**12.36 (6.82, 17.90)**	**<0.01**	0.10
**Omega-3**												
Omega-3	20	0.60 (-0.90, 2.11)	0.39	0.90	19	0.49 (-0.46, 1.44)	0.27	0.85	21	1.40 (-0.18, 2.98)	0.08	0.58
**Trans FA**												
Total Trans FA	20	-0.23 (-1.01, 0.55)	0.53	0.92	19	-0.18 (-0.68, 0.33)	0.45	0.90	21	-0.02 (-0.97, 0.93)	0.97	1.00
Trans 16:1	20	7.06 (-17.97, 32.09)	0.54	0.93	19	5.15 (-14.40, 24.71)	0.57	0.93	21	-16.33 (-44.47, 11.82)	0.23	0.81
Trans 18:1	20	-0.26 (-1.13, 0.61)	0.52	0.92	19	-0.21 (-0.78, 0.35)	0.41	0.90	21	0.11 (-0.95, 1.16)	0.83	0.99
Trans 18:2	20	-1.33 (-7.33, 4.67)	0.63	0.96	19	-0.52 (-4.44, 3.40)	0.77	0.98	21	-2.68 (-9.66, 4.31)	0.41	0.90
CLA cis9 trans11	20	5.29 (-10.09, 20.66)	0.46	0.90	19	1.47 (-9.93, 12.87)	0.78	0.98	21	-9.81 (-27.34, 7.72)	0.24	0.81
CLA trans10 cis12	20	16.56 (-55.41, 88.53)	0.62	0.96	19	-9.79 (-58.91, 39.33)	0.66	0.96	21	-22.28 (-107.71, 63.14)	0.57	0.94
**CLA**												
CLA 18 2	20	5.19 (-8.27, 18.65)	0.41	0.90	19	0.51 (-9.45, 10.48)	0.91	1.00	21	-6.19 (-22.22, 9.84)	0.41	0.90

β = unstandardized regression coefficient; 95% CI = 95% confidence interval; p = unadjusted p-value; q = Benjamini–Hochberg false discovery rate (FDR)-adjusted p-value; n = sample size. Bold values indicate q < 0.05. All models were adjusted for sedentary behaviour, age, sex, BMI, alcohol intake, family history of high cholesterol, NSAID use, cholesterol medication use, smoking status, and blood pressure treatment. Imm = Immediate; h = hours.

Table S10. Sensitivity of the association between habitual MFA 20:1 (eicosenoic acid) intake and 48-hour post-race inflammatory markers across progressively adjusted model specifications, with leave-one-out and bootstrap stability checks on the fully adjusted model.

Model specification	Covariates added	n	β (95% CI)	p
IFN-γ, 48-hour post-race				
Energy-adjustment only	Energy intake	23	11.16 (5.86, 16.46)	<0.001
+ demographics	+ age, sex, BMI	20	11.27 (5.95, 16.59)	<0.001
+ lifestyle	+ sedentary behavior, alcohol, smoking	20	11.38 (5.32, 17.43)	0.001
Full model (primary model)	+ family history of hypercholesterolemia, habitual NSAID use, cholesterol medication, antihypertensive treatment	20	13.66 (8.94, 18.38)[a]	<0.01
TNF-α, 48-hour post-race				
Energy-adjustment only	Energy intake	24	5.46 (1.02, 9.91)	0.018
+ demographics	+ age, sex, BMI	21	6.28 (1.53, 11.03)	0.013
+ lifestyle	+ sedentary behavior, alcohol, smoking	21	6.77 (1.83, 11.70)	0.011
Full model (primary model)	+ family history of hypercholesterolemia, habitual NSAID use, cholesterol medication, antihypertensive treatment	21	9.14 (6.43, 11.86)[b]	<0.01

Note. Each model adds covariates cumulatively to the row above. Leave-one-out stability (full/primary model): IFN-γ β estimates ranged from 12.42 to 15.77 (full-sample β=13.66, mean=13.68, SD=0.73); TNF-α β estimates ranged from 8.27 to 10.76 (full-sample β=9.14, mean=9.10, SD=0.54). The same participant was most influential in both models, and removing them increased rather than decreased the magnitude of the association.

[a] Bootstrap resampling (1000 iterations; 367/1000 valid resamples) of the fully adjusted IFN-γ model produced a wider 95% CI (2.61, 27.61) than the asymptotic estimate, indicating the asymptotic standard errors likely understate true sampling uncertainty; the resampled interval remained entirely positive.

[b] Bootstrap resampling (1000 iterations; 340/1000 valid resamples) of the fully adjusted TNF-α model produced a wider 95% CI (2.27, 13.74) than the asymptotic estimate, also remaining entirely positive.

Table S11. Association of Energy- and Covariate-Adjusted MFA 20:1 (Eicosenoic Acid) Intake with Post-Race Inflammatory Cytokines: Primary Models and Sensitivity Analysis Further Adjusting for Training Volume

	n	β (95% CI)	p-value
		IFN-γ, 48-hour post-race	
Primary Model [a]	20	**13.66 (8.94, 18.38)**	**<0.01**
Sensitivity Analysis [b]	20	**12.95 (6.94,18.96)**	**0.0003**
		TNF a, 48-hour post-race	
Primary Model[a]	21	**9.14 (6.43, 11.86)**	**<0.01**
Sensitivity Analysis [b]	21	**8.43 (3.64, 13.21)**	**0.002**

Abbreviations: CI, confidence interval; IFN-γ, interferon-gamma; MFA, monounsaturated fatty acid; SD, standard deviation; TNF-α, tumor necrosis factor-alpha.

a Primary models were adjusted for total energy intake, sedentary behavior, age, sex, BMI, alcohol intake, family history of hypercholesterolemia, NSAID use (habitual), cholesterol medication use, smoking status, and antihypertensive treatment using the multivariate residual method.

b Sensitivity analyses further adjusted for weekly running mileage in the multivariate residual adjustment model. All other covariates were retained.

Table S12. Sensitivity analysis of the association between habitual MFA 20:1 intake and 48-hour post-race IFN-γ and TNF-α, additionally adjusting for post-race recovery-period behaviors.

Model	n	β (95%CI)	p-value
IFN-γ, 48-hour post-race			
Primary model[a]	20	13.66 (8.94,18.38)	<0.01
+ post-race NSAID use[b]	20	12.37 (7.73,17.00)	<0.001
+ below-average sleep night after race[b]	18	14.26 (7.08, 21.43)	0.003
+ finishing time[b]	20	13.09 (8.32, 17.85)	<0.001
+ recovery-period alcohol (continuous, g/day) [b]	18	8.91(2.90, 14.92)	0.012
TNF-α, 48-hour post-race			
Primary model[a]	21	9.14 (6.43, 11.86)	<0.01
+ post-race NSAID use[b]	21	8.79 (5.80,11.7)	<0.001

Model	n	β (95%CI)	p-value
+ below-average sleep night after race[b]	19	8.93 (5.25,12.61)	<0.001
+ finishing time[b]	21	9.03 (6.07, 11.99)	<0.001
+ recovery-period alcohol (continuous, g/day)[b]	19	9.6 (04.58, 14.62)	0.003

Note. [a]Primary model adjusted for total energy intake, sedentary behavior, age, sex, BMI, alcohol intake, family history of hypercholesterolemia, NSAID use (habitual), cholesterol medication use, smoking status, and antihypertensive treatment, using the residual method for energy/covariate adjustment of habitual MFA 20:1 intake. [b]Sensitivity models add one post-race recovery-period covariate at a time to the fully adjusted primary model. NSAID use after race and sleep deviation the night following the marathon were assessed via a follow-up questionnaire administered at the 48-hour visit. Recovery-period alcohol intake was measured using 24-hour dietary recalls collected at 48-hour post-race collected using the validated Automated Self-Administered 24 h Dietary Assessment Tool (ASA24).[50] Reduced n in sensitivity models reflects additional listwise deletion for missing recovery-period covariates.
